# Corticosterone effects induced by stress and immunity and inflammation: mechanisms of communication

**DOI:** 10.3389/fendo.2025.1448750

**Published:** 2025-03-20

**Authors:** Jingyu Xu, Baojuan Wang, Haiqing Ao

**Affiliations:** ^1^ School of Public Health and Management, Guangzhou University of Chinese Medicine, Guangzhou, China; ^2^ Department of Reproductive Medicine, First Teaching Hospital of Tianjin University of Traditional Chinese Medicine, Tianjin, China; ^3^ National Clinical Research Center for Chinese Medicine Acupuncture and Moxibustion, Tianjin, China

**Keywords:** stress, corticosterone, immunity, inflammation, mechanism

## Abstract

The body instinctively responds to external stimuli by increasing energy metabolism and initiating immune responses upon receiving stress signals. Corticosterone (CORT), a glucocorticoid (GC) that regulates secretion along the hypothalamic-pituitary-adrenal (HPA) axis, mediates neurotransmission and humoral regulation. Due to the widespread expression of glucocorticoid receptors (GR), the effects of CORT are almost ubiquitous in various tissue cells. Therefore, on the one hand, CORT is a molecular signal that activates the body’s immune system during stress and on the other hand, due to the chemical properties of GCs, the anti-inflammatory properties of CORT act as stabilizers to control the body’s response to stress. Inflammation is a manifestation of immune activation. CORT plays dual roles in this process by both promoting inflammation and exerting anti-inflammatory effects in immune regulation. As a stress hormone, CORT levels fluctuate with the degree and duration of stress, determining its effects and the immune changes it induces. The immune system is essential for the body to resist diseases and maintain homeostasis, with immune imbalance being a key factor in the development of various diseases. Therefore, understanding the role of CORT and its mechanisms of action on immunity is crucial. This review addresses this important issue and summarizes the interactions between CORT and the immune system.

## 1 Introduction

During various physiological, psychological, and social stress events—such as those arising from unhealthy habits, pessimistic cognition, work difficulties, and interpersonal conflicts—the body undergoes a series of physiological stress reactions (1–3). It’s important to note that these stressors are responsible for inducing physiological changes. In today’s society, stress events are commonplace. While moderate stress can enhance the body’s ability to cope with social challenges, prolonged stress can detrimentally affect the functioning of various bodily tissues, with the degree of damage increasing over time (4, 5). In response to stress, the body employs intricate coping mechanisms involving neural transmission, sequential activation of signaling molecules, and interactions among different bodily systems, ultimately leading to changes in physiological representation and behavioral patterns. Although the body can adapt to stressors, this adaptation may come at the expense of health. Currently, numerous diseases have been associated with chronic stress, including anxiety, depression, cognitive impairment, inflammatory gastrointestinal diseases, metabolic syndrome, autoimmune disorders, and infertility (6–10). It can be seen that chronic stress will disrupt system function from multiple perspectives. And in the later stages of the disease, mental disorders such as anxiety and depression often co occur with peripheral lesions. Such as cardiovascular disease, gastrointestinal dysfunction, autoimmune diseases, and infertility. According to statistics, the lifetime prevalence of anxiety disorder is 5% -13% (11), and in a very few countries, such as the United States, it can reach 34% (12). Shorey S’s study shows that the global prevalence of self-reported depression is 34%, of which the incidence rate of major depressive disorder (MDD) is 8% and the lifetime prevalence is 19% (13). And these proportions are still increasing. During the SARS-CoV-2 pandemic, the global emotional burden increased, with an increase of 76.2 million cases of anxiety disorder and approximately 18.6% of people experiencing anxiety being accompanied by moderate to severe depression (14, 15). Krittanawong C’s meta-analysis showed that depression increases the risk and mortality of cardiovascular diseases, such as congestive heart failure and myocardial infarction (16). And Brock J’s systematic review suggests that depression increases disability and mortality in rheumatoid arthritis (RA) (17). Clinical data shows that similar immune mechanisms (excessive secretion of pro-inflammatory cytokines) often lead to comorbidity between the two and mutually promote their onset. The incidence rate of depression in RA is 2-3 times that of the general population, and about 16.8% of RA patients suffer from depression (18). In addition, Indira R’s review provides a detailed report on the association between depression and sexual dysfunction (19), with sexual dysfunction observed in 63% of MDD patients (20). There are also epidemiological clues that indicate a decrease of sex hormone levels in patients with depression (21). Thus, stress-induced injury is systemic in nature. There are common pathological mechanisms among various diseases associated with stress, leading to their interdependence. Owing to the complexity of its mechanisms and its widespread detrimental effects, making it a prominent focus of research. Meanwhile, this is also a crucial step in exploring the etiology and therapeutic targets of diseases.

Corticosterone (CORT), a type of glucocorticoid (GC), is a product of the hypothalamic-pituitary-adrenal (HPA) axis. It is rapidly secreted and regulated under neural control and plays a crucial role in stress adaptation due to its wide range of hormonal properties (10). In long-term stress conditions, the feedback mechanism of the HPA axis gradually falters, leading to an increase in serum basal CORT levels (22, 23). This elevation has been associated with various diseases. Chronic mild systemic inflammation serves as an important pathway mediating disease occurrence, characterized by increased levels of pro-inflammatory cytokines in serum, dysfunction of bone marrow and lymph nodes, and inflammatory damage to brain neurons. CORT has been shown to exert pro-inflammatory effects (24–26). However, stress response is a complex physiological and pathological process. During the initial stages of acute stress, the rapid elevation of CORT concentration demonstrates anti-inflammatory effects in managing acute events (27). The level of CORT depends on the severity and duration of exposure to the stressor, as well as the traumatic effects of stress (28, 29). As a mediator between stress and bodily responses, the dual role of CORT has attracted considerable research attention. The concentration, duration, and mode of action of serum CORT are key determinants influencing different effects (30).

The immune system serves as the body’s protective mechanism and can recognize and eliminate foreign antigens as well as mutated or aging cells within the body (31). Its primary role is to maintain organismal internal environment homeostasis, with bodily damage and repair processes relying on its functionality. Thus, when confronting and managing stressful situations, the immune system’s function is indispensable. The interaction between the effects of CORT and the immune system is essential in determining the body’s state and trajectory during stress (23). Studies have demonstrated that depression can compromise the immune system, increasing the susceptibility to infection (32, 33). Prolonged elevation of serum basal CORT levels continuously activates the immune system, disrupting the body’s homeostasis and leading to various forms of damage (10, 34, 35). Chronic inflammation, widely recognized as a mechanism of injury, is frequently implicated in this process (36, 37). Despite its defensive role, chronic inflammation also contributes to tissue damage. Nevertheless, research has shown that even a slight increase in CORT levels can alleviate oxidative damage and enhance innate immunity (38). Therefore, understanding the relationship between CORT concentration, its dual effects, and subsequent immune system signaling regulation is crucial, given its significance as a key stress hormone in peripheral circulation.

**Figure 1:**
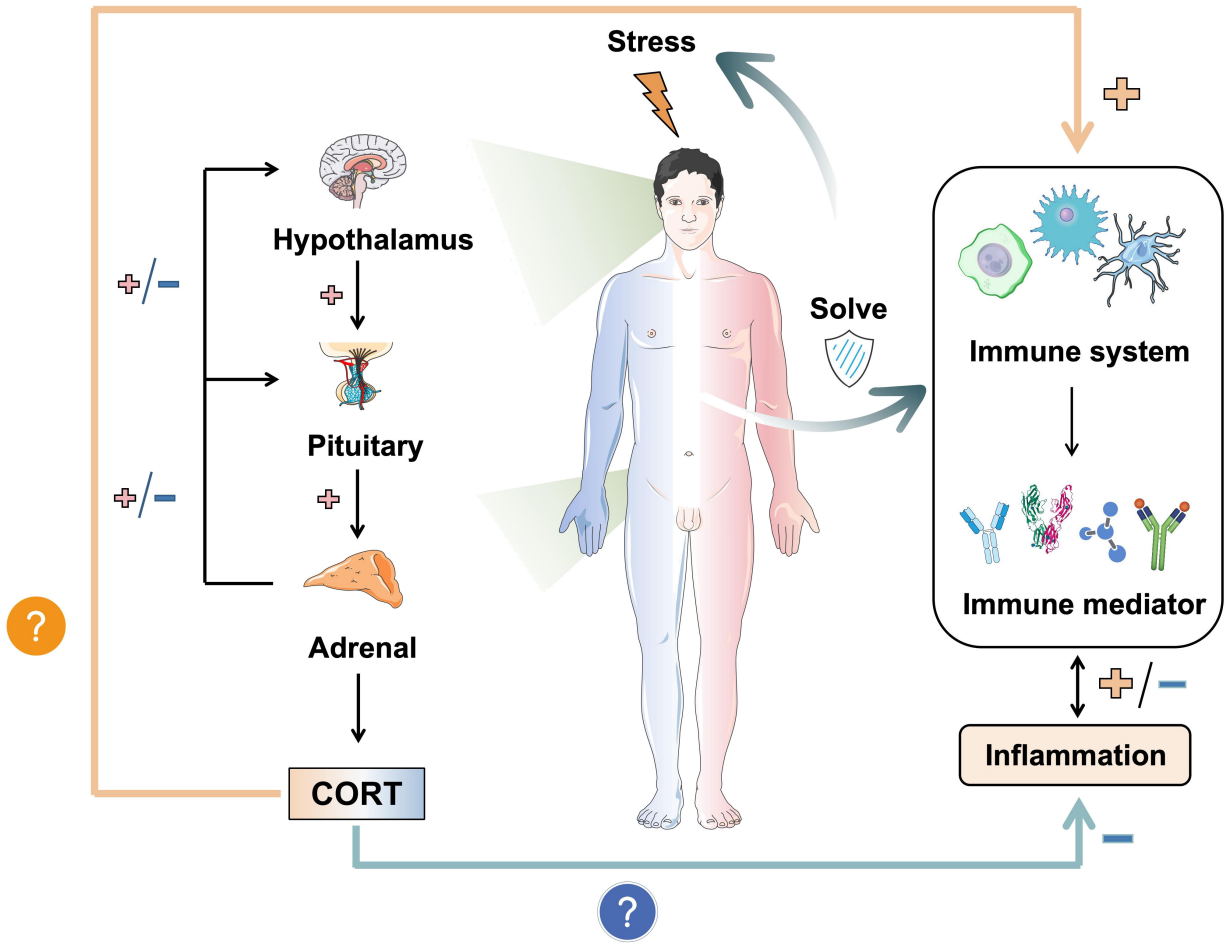
The dual mechanism of stress-induced CORT on immunity and inflammation. Stress induces the release of CORT by activating the HPA axis. It is worth noting that the immune effects of CORT are dual, namely anti-inflammatory and pro-inflammatory. It depends on the duration of stress and the mode of action of CORT. How CORT interacts with the immune system and correlates inflammation and disease is the focus. In this figure, →: Action site. +: Positive feedback signal transmission/activation. -: Negative feedback signal transmission/suppression.

The impact of stress response on the body is profound, with the HPA axis and the immune system serving as the primary mechanisms for coping with challenges. These systems are closely interrelated and represent important pathways through which stress can induce various biological injuries. CORT, a hormone produced by the HPA axis, directly interacts with the immune function of the body’s tissues in the bloodstream, underscoring the significance of its regulatory mechanism. The level of CORT serves as a tangible indicator of stress in the body, with its effects being closely linked to susceptibility to various diseases through modulation of the immune system and mediation of inflammation development (39). Therefore, the purpose of this review is to elucidate and analyze the relationship between CORT levels and immune regulation and to summarize the biological mechanisms through which stress impacts the immune system. The research question is shown in **Figure 1**. Given the intimate connection between the immune system and various tissues throughout the body, this review aims to facilitate the exploration of the pathogenesis of systemic diseases triggered by stress in the future and to clarify the intricate relationship between stress and the body at the cellular and molecular levels.

## 2 Changes in corticosterone levels and their immune effects

### 2.1 The dual identity of CORT concentration controlled by stress and HPA axis

#### 2.1.1 Short term stress (acute stress)

CORT, as the primary hormone driving the adverse effects of chronic stress on the body, holds significant importance in both the immune system response and stress response (40). Numerous studies have indicated that the HPA axis remains active, and circulating CORT concentrations increase in individuals with depression (41). Serving as the swiftest neuroendocrine regulatory mechanism for stress response, the HPA axis’s failure in feedback mechanisms leads to chronic damage through inflammation (42). Repeated administration of CORT stimulation in animals has been observed to lead to HPA axis dysfunction, neuronal damage, cognitive decline, and memory impairment through the P2X7/NF-κB/NLRP3 signaling pathway (43). Data from early studies showed that, it is generally accepted that the baseline plasma concentration of CORT in rats ranges from 50-100 ng/ml (44), while in stressed rats, it can range from 120-425 ng/ml (45, 46). CORT levels simulating acute stress stimulation usually exceed 15-20 ng/ml (47), and *in vivo*, CORT concentrations of 1-5 nM can bind with GC receptors (GR) to exert effects (48).

The mechanism of CORT production in the central nervous system has been extensively studied and summarized (49, 50). Recent research has revealed that the activation of Agouti-related protein (AgRP) neurons, which are related to autophagy and energy metabolism, also promotes CORT production (51). Research indicates that acute stress triggers the activation of AgRP neurons, leading to the expression of neuropeptide Y (NPY), which then promotes presynaptic inhibition of GABAergic neurons expressing NPY1R and activates CRH neurons in the paraventricular nucleus (PVN) of the hypothalamus, thereby stimulating the HPA axis and increasing circulating CORT levels. Subsequently, negative feedback regulation of the HPA axis inhibits AgRP neuron activation and CORT secretion. The increase in CORT caused by acute stress is swiftly suppressed to a resting value due to this negative feedback regulation. However, long-term chronic stress accompanied by HPA axis feedback failure gradually elevates basal CORT levels, which cannot be effectively reduced. At this stage, CORT activates immunity and triggers inflammation as the main effect (52, 53).

Observations of cell states reveal that CORT exhibits two immune effects at different concentrations. Emaya et al. (54) investigated the impact of GP120, a neurotoxic viral glycoprotein, on human microglia (HMC3). In the absence of GP120 treatment, CORT at concentrations of 32, 100, and 320 nM activated HMC3 cell activity, with the most pronounced effect observed at 100 nM. Following GP120 treatment, 100 nM CORT effectively attenuated GP120-induced neuroinflammatory damage in HMC3 cells within 24 hours, whereas the effects of 32 and 320 nM CORT were not significant. This highlights the anti-inflammatory role of CORT in acute infections. Moreover, similar to Emaya et al.’s findings (55), the simultaneous addition of GP120 and 1 μM CORT mitigated GP120-induced neurotoxicity in microglia. In the early stage of GP120 infection, an instantaneous increase in CORT concentration exerts immunosuppressive effects by promoting macrophage phagocytosis activation, clearing pro-inflammatory cells and debris, and inhibiting the production of neurotoxic cytokines within glial cells. However, following pre-treatment with 1 μM CORT for 24 hours and subsequent addition of GP120, the neurotoxic effect of GP120 in microglia was enhanced by 15%. Long-term exposure to increasing CORT concentrations promotes inflammation, indicating that the effect of CORT is related not only to its absolute concentration but also to whether the concentration is stable or fluctuating. The timing of CORT administration is also crucial. Pre-treatment with CORT before infection typically has a dominant cytotoxic effect, whereas post-infection CORT treatment demonstrates an anti-inflammatory effect. This suggests that prolonged elevated CORT continuously activates and depletes the immune system, leading to more severe damage when encountering additional stressors.

#### 2.1.2 Long term stress (chronic stress)

Prolonged stress can lead to chronic inflammation, with continuous production of inflammatory cytokines (56). The internal environment influenced by age also affects the immune regulatory effect of CORT, and age is a significant predictor of the severity of stress-induced damage (57), often correlating with the duration of stress exposure. The aging phenotype is essential, as aging can impair both the activation of the HPA axis induced by acute stress and the feedback regulation capability of the HPA axis (58). In elderly mice, the increase in CORT levels after acute stress is less pronounced than in young mice, and CORT levels remain elevated compared to the resting value at 4 hours. In contrast, the HPA axis feedback regulation in young mice is more sensitive, with CORT peaking at 2 hours and returning to baseline by 4 hours (51). Changes in CORT levels during stress are related to the function of the HPA axis. In young mice, normal HPA axis feedback inhibits the effects of high-level CORT, while older mice exhibit poorer stress responses and are more susceptible to damage from prolonged CORT exposure. Cellular aging mediates the response to stressors, involving mechanisms such as the HPA axis hormones, the sympathetic and parasympathetic nervous systems, thymic hormones, and pineal melatonin (59). Therefore, the HPA axis is a key regulator for the body’s adaptation to stress. Dysfunction of the HPA axis can synergistically lead to various stress-dependent diseases through neural, immune, and endocrine pathways (60, 61). Individual characteristics are crucial for identifying those with increased vulnerability to stress (62). During acute stress, CORT levels increase but quickly return to baseline, with this range of increase and decrease diminishing with age (63). This indicates that the ability to handle stress deteriorates with age, increasing susceptibility to damage. Additionally, sensitivity to stress depends on factors such as the internal environment, genetic diversity, and gender (64).

### 2.2 Immune activation and immunosuppression caused by CORT

The behavioral changes induced by fluctuations in cyclic CORT levels exhibit a holistic nature. However, the effects of CORT may vary locally depending on specific tissues and cells (65). Factors such as enzyme activity, expression levels of GR (encoded by Nr3c1), receptor variations, and local cellular signaling interference (e.g., NF-κB, CREB, STAT signals) may influence CORT utilization (66–68). The difference between immune enhancement and inhibition caused by stress appears to be mediated by the duration, intensity, and observed immune components of the stressor (69). Additionally, sustained stress characterized by inflammatory aging leads to immune system depletion, resulting in overall immune suppression (70, 71). The duration of stress exposure is an important factor in guiding the immune response. Sarjan et al. were the first to examine the effects of stress exposure duration on the immune system (72). Animal experiments showed that prolonged exposure significantly decreased the count and activity of immune cells (myeloid cells and lymphocytes), bone marrow stem cells, blood immunoglobulin, and IL-12 levels. Conversely, 3β-hydroxysteroid dehydrogenase (3β-HSD) activity, circulating immune complexes (CIC), and IL-10 levels increased with prolonged exposure. Cell experiments confirmed the concentration-dependent immunosuppression of CORT, with CORT-induced cell death being the primary cause of immune dysfunction. Short stress exposure leads to a faster recovery, whereas sustained stress may cause irreversible damage.

**Figure 2:**
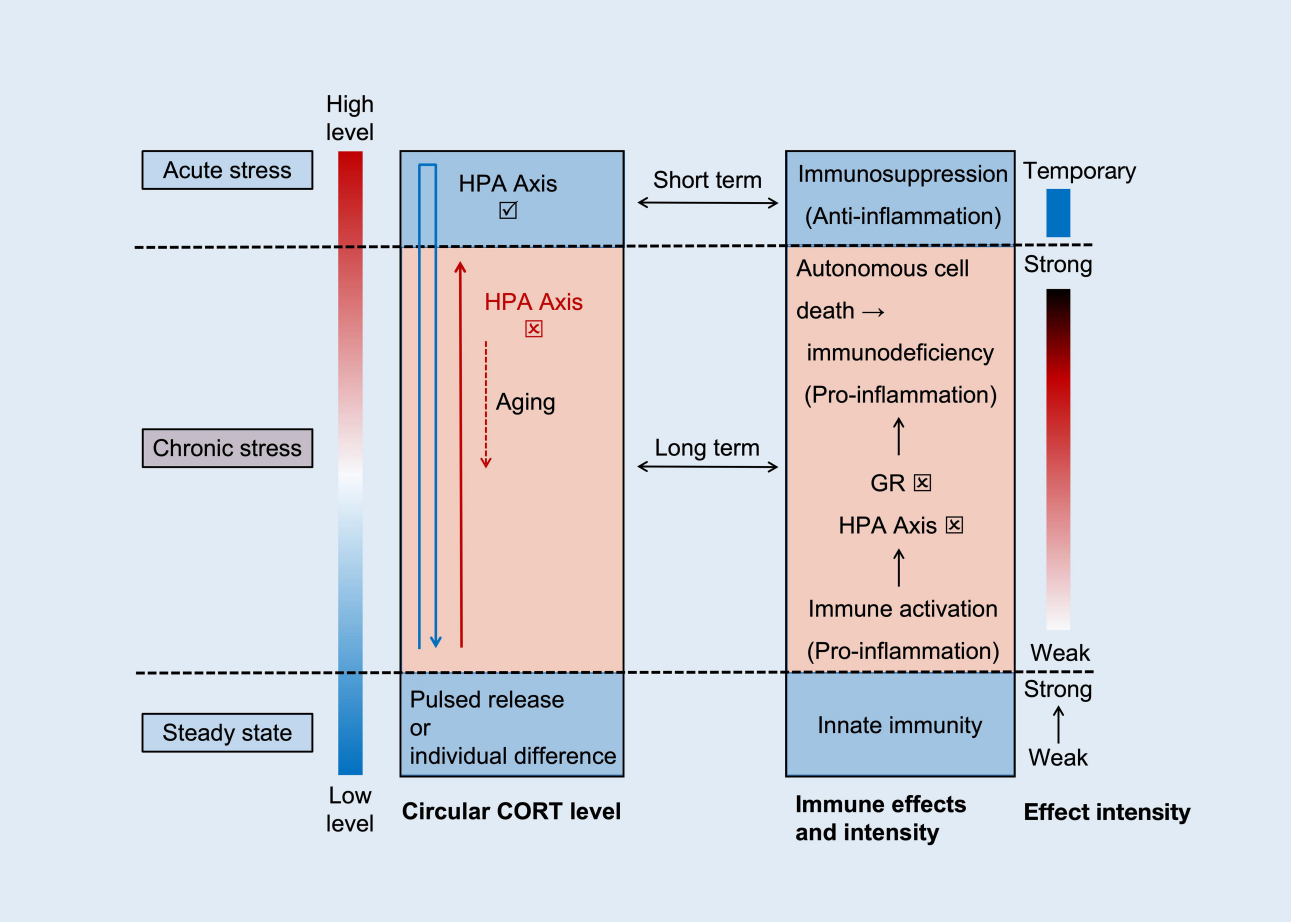
The relationship between CORT cycle level and immunity. The levels and functions of CORT are manifested in three distinct scenarios, namely steady state (normal level), acute stress (rapid high level and then recovery), and chronic stress (slow increase - sustained high level - slow decrease). Within the normal threshold, CORT levels are positively correlated with innate immune function. In acute stress, CORT rapidly increases (blue arrow), with anti-inflammatory effects being the dominant effect. And after the stress ends, restore stability based on the negative feedback function of HPA axis. Both are blue background boxes, representing physiological status. In chronic stress, CORT slowly increases and remains at a high level (red solid arrow), with pro-inflammatory effects being the dominant effect. As time goes on, dysfunction of the HPA axis and GR further exacerbates the inflammation and promotes cell death. Eventually, with aging, CORT slowly decreases (red dashed arrow), and immune suppression at this point is the result of immune exhaustion.

Regarding immune activation, even an increase in CORT concentration within a physiological range (microstimulation) can activate immune function. Innate immunity is primarily affected by its mechanism, which is related to the production of pro-inflammatory mediators. When the stressor is removed, CORT is quickly suppressed by the HPA axis to its resting value. Vágási CI conducted a study on maintaining CORT levels within the physiological baseline range (38). The intervention involved subcutaneously implanting drug particles containing 2 mg CORT in sparrows (degraded after about 2 months), raising plasma CORT concentration to approximately 8.5 ng/ml. One month post-implantation, this intervention was found to significantly increase the humoral components of innate immunity in sparrows, including natural antibodies and complement levels (measured by hemolysis hemagglutination assay). However, no significant changes were observed two months post-implantation, possibly due to drug particle degradation. Previous studies have shown similar results (73, 74), indicating that short-term stress exposure visibly enhances the innate immune system. While enhancing immunity may seem beneficial, it comes at the cost of increased material and energy metabolism. Animals treated with CORT in this study showed weight loss and poor hair quality despite enhanced innate immunity. Immune activation increases energy consumption and metabolite production, such as oxides and acidic substances, which can induce cell damage and further stimulate inflammation (75). Therefore, the level and duration of CORT affect the body’s ability to metabolize and decompose toxins. Prolonged stress and the inability to reduce CORT concentration can cause functional changes, disrupting the balance between damage and repair. Sustained stress exacerbates damage and may lead to disease induction. The relationship between circulating CORT levels and immunity is illustrated in **Figure 2**.

### 2.3 The immune effects of stress and CORT

#### 2.3.1 Innate immunity and acquired immunity

**Figure 3:**
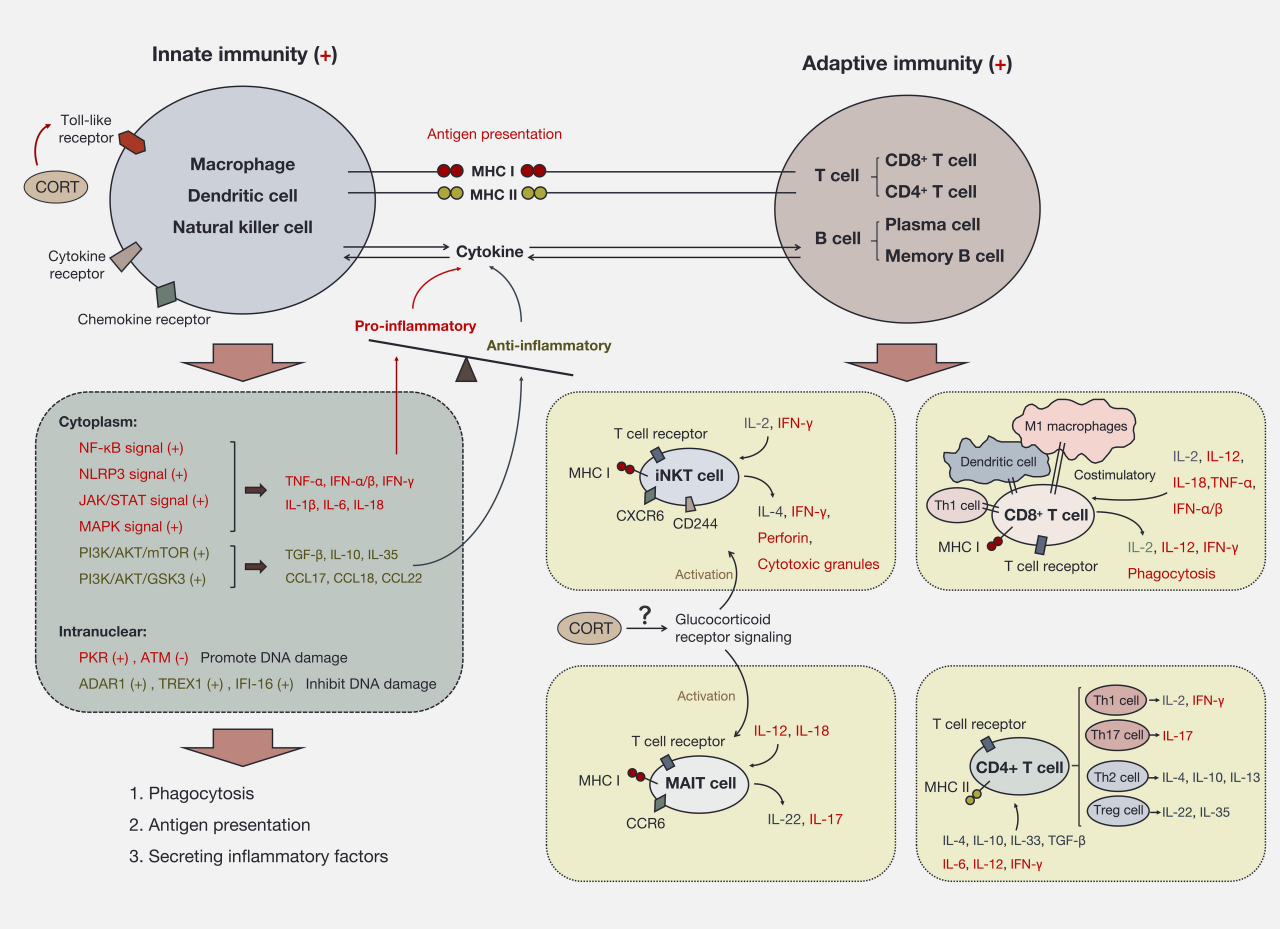
Immune activation and immune imbalance induced by CORT. CORT activates the immune system via Toll-like receptors. Innate immune cells are the first responders to CORT. The intracellular signaling pathways triggered by CORT produce two main effects, on the one hand, the secretion of pro-inflammatory factors to enhance immune activity. On the other hand, the secretion of anti-inflammatory factors to protect the cells themselves and to restrain excessive immune responses. The sustained high-level CORT further activates adaptive immune cells and expands immune activity. At this time, a large number of immune cells participate in the circumstance and transmit signals to each other through inflammatory factors. If CORT persists as an upstream signaling molecule, it will continue to deplete the immune system, leading to the exacerbation and spread of inflammation.

Most immune responses to stress are initiated through innate immune pathways. Toll-like receptors (TLRs) on the immune cell membrane are essential for CORT’s influence on the immune response. In macrophages, dendritic cells (DCs), and natural killer (NK) cells, TLR activation promotes intracellular NF-κB phosphorylation and nuclear translocation, enhancing antigen presentation, phagocytosis and the production of various pro-inflammatory cytokines, such as IL-1β, IL-6, TNF-α, IFN-γ, and IL-18 (76, 77). This process also activates caspase-1, further promoting inflammatory signaling (78). In acute stress situations, such as on the skin’s surface, cytokines and chemokines recruit macrophages and NK cells to the site to prevent the spread of pathogens (79). Typically, innate immunity is utilized to counter external stress. If the response concludes at this stage, innate immune cells will not engage T cells. Instead, they present antigens to B cells to establish immunological memory (memory B cells) and produce antibodies (plasma cells) for future stress conditions (80). Importantly, long-term elevated CORT due to chronic stress is believed to cause immune cells to continuously receive “battle” signals, perpetually activating both innate and acquired immunity (81). Cytokines produced by innate immune cells (i.e., NK cells and macrophages) create signaling connections with immune cells (T cells and B cells) involved in acquired immune responses in the peripheral blood circulation, with antigen presentation further enhancing this connection. Antigen-presenting cells expressing major histocompatibility complex class I (MHC I) primarily activate CD8^+^ T cells, which are pivotal for cellular immunity by engaging in the phagocytosis of antigens (82). Conversely, antigen-presenting cells expressing MHC II mainly activate CD4^+^ T cells (helper T cells) (82, 83). Subsequently, these activated CD4^+^ T cells can initiate the activation of B cells, prompting their differentiation into plasma cells responsible for antibody secretion, thereby fostering humoral immunity (84). Additionally, helper T cells polarize into different phenotypes (Th1/Th2 and Th17/Treg) and secrete various cytokines to participate in cellular immunity (85). To cope with stress-mediated damage, restoring balance within the organism requires synergistic interactions between the innate and acquired immune systems. The regulation of inflammation involves both positive and negative feedback mechanisms that coexist. For example, IFN-α/β can stimulate Th1 cells, DCs, and M1 macrophages to co-stimulate MHC I, enhancing cytotoxic T lymphocyte (CTL) activity and the expression of IL-2, IL-12, and IFN-γ, thereby promoting inflammation (86–88). Conversely, IFN-α/β can stimulate Th2 cells, Treg cells, and M2 macrophages to secrete IL-4, IL-10, and PD-1, which suppress the expression of pro-inflammatory factors and adhesion molecules such as TNF-α, IL-1β, and IL-8 and in turn downregulates MHC I expression to suppress CTL activation, thereby achieving immune control (89–93). These cytokines, as non-specific regulatory factors, affect most immune responses (7). Thus, immune activation and inhibition can coexist. The immune response triggered by long-term elevated CORT adapts through dynamic shifts in immune cell populations to achieve a new steady state, such as M1/M2 macrophages, Th1/Th2 cells, and Th17/Treg cells. As shown in **Figure 3**.

#### 2.3.2 Invariant T cells

In addition to the traditional T cells mentioned above, invariant T cells (iT cells) have become a significant focus of research in immune regulation. iT cells are congenital T cells, including invariant natural killer T cells (iNKT cells) and mucosa-associated invariant T cells (MAIT cells). Functionally, they are considered bridges between innate and acquired immunity (94, 95). iNKT cells are characterized by expressing both NK cell surface marker CD244 and chemokine receptor CXCR6, as well as T cell surface marker TCR α/β (96). They can be activated by MHC I antigen presentation and by pro-inflammatory factors IL-2 and IFN-γ (97, 98). iNKT cells primarily recognize lipid antigens (99). Upon activation, they can secrete perforin and granzymes or utilize the Fas/FasL pathway to kill cells, participating in immune responses. Additionally, they can secrete IFN-γ or IL-4 to induce Th1 or Th2 differentiation, thereby playing a regulatory role (100, 101). MAIT cells also express TCR, which is activated by MHC I antigen presentation (TCR-dependent pathway) or by IL-12 and IL-18 (non-TCR-dependent pathway) (102). Upon activation, IL-18R and CCR6 are overexpressed on the cell membrane (102, 103). MAIT cells can secrete both the pro-inflammatory factor IL-17 and the anti-inflammatory factor IL-22, with their dual immune regulatory functions showing tissue specificity (103). MAIT cells also possess antioxidant functions, which may limit neuroinflammation and ensure cognitive function (104). However, insufficient or excessive MAIT cellular activity can induce autoimmune diseases, inflammatory diseases, and allergic diseases through dysbiosis of the microbiota (105–107). Previous studies have shown that stress promotes Th2 phenotype bias and inhibits Th1 activation by NE, NPY, and CORT (108–110). Recent studies have reported that chronic stress impairs the function of iT cells, demonstrating a mixed feature of selectively inducing the production of pro-inflammatory and anti-inflammatory cytokines (102, 111).

Long-term stress reduces the expression of TCRα/β, CD28, and inducible T cell costimulator (ITCOS) on the surface of iNKT cells, thereby decreasing their ability to secrete IL-4 and IFN-γ (102). IL-4 and IFN-γ are key factors in promoting Th2 and Th1 differentiation, respectively (112). Following stress, cytokine analysis revealed decreased serum levels of IL-2, IL-5, IL-13, Eotaxin, GM-CSF, IP-10/CXCL10, MCP-1/CCL2, RANTES/CCL5, and TNF-α, while the levels of IL-1α, IL-1β, MIP-1α/CCL3, and MIP-3α/CCL20 increased. In iNKT cells, the expression of IL-2, IL-5, IL-12, and IL-13 decreased, whereas IL-10, IL-23, and IL-27 levels increased. Cytokines and chemokines unaffected by stress include G-CSF, IL-6, IL-7, IL-9, IL-15, IL-17E/IL-25, IL-17F, IL-21, IL-22, IL-28B/IFNL3, IL-31, IL-33, KC/CXCL1, LIF, LIX/CXCL5, M-CSF, MIG/CXCL9, MIP-1β/CCL4, MIP-2/CXCL2, TGF-β1, TGF-β2, TGF-γ3, and VEGF. Therefore, the regulation of iNKT cells on inflammatory factors under stress is twofold. Upstream of the iNKT cell response, increased levels of the anti-inflammatory protein glucocorticoid-induced leucine zipper (GILZ) were detected, confirming that the response was dependent on GR signaling rather than sympathetic nervous system (SNS) signaling, as no change in SNS neurotransmitter receptor expression was observed (102). GILZ is a known transcriptional target for GR activation (113). Studies have shown reduced transcription levels of genes related to iNKT cell effector functions, including *Cd40l*, *Il18rap*, *Egr2*, *Irf4*, *Nfatc3*, *Tbx21*, *Ifng*, *Il4*, *Gzma*, *Tnf*, *Tnfrsf9* and *Tnfsf10*, suggesting that stress can inhibit iNKT cell function through GR signaling (102). MAIT cells also rely on the GR pathway to activate defense mechanisms under stress. Similar to iNKT cells, MAIT cells overexpress CD127 and reduce the secretion of IL-4 and IFN-γ to attenuate Th1 and Th2 responses (102). The above indicates that stress suppresses the immune response by impairing iT cell function. However, due to the dual role of iT cells in regulating both pro-inflammatory and anti-inflammatory cytokines and their ability to differentiate into different T cell phenotypes, further exploration of their role in various tissues is necessary. Additionally, the upstream mechanisms of GR signaling and CORT regulation require further investigation through cell experiments.

#### 2.3.3 DNA damage response

Under chronic stress conditions, the inflammatory response during immune activation can also affect DNA damage repair and epigenetic modification (114). In the innate immune response, the activation of Pattern Recognition Receptors (PRRs) on the cell membrane leads to long-term activation of double-stranded RNA-dependent protein kinase (PKR), resulting in the inactivation of the DNA repair kinase ATM (part of the PI3K protein kinase family) and the phosphorylation of p65 NF-κB, thereby promoting IFN-γ synthesis (86). PKR activation also stimulates the NLRP3 inflammasome, enhancing the synthesis of IL-1β and IL-18, a process that can be inhibited by p58^IPK^ (115). Additionally, the RNA editing enzyme ADAR1, the nucleic acid repair exonuclease TREX1, and the interferon-induced nuclear protein IFI-16 are activated to ensure DNA repair and prevent abnormal activation of interferons (116–118). ADAR1 has been found to prevent the activation of the receptor MDA5/PKR by A-RNA, thereby inhibiting IFN production and translation, and exerting immunosuppressive effects. Additionally, ADAR1 can inhibit RIPK3/MLKL-dependent programmed cell necrosis by blocking Z-RNA activation of ZBP1 (119, 120). Changes in transcription levels in the nucleus are associated with the extracellular JAK/STAT signaling pathway, PI3K/AKT/GSK3, and PI3K/AKT/mTOR signaling pathways (121, 122). Alongside the inflammatory response, the anti-inflammatory response is also regulated. For instance, the PI3K/AKT signaling pathway controls inflammation by upregulating the expression of IL-10-induced genes, thus antagonizing the cytotoxic effects of pro-inflammatory factors TNF-α, IL-1β, IL-6, and IL-8. Additionally, the expression of Caspase-3, Caspase-8, and Caspase-9 is downregulated to reduce apoptosis (123–125). In the process of adapting to stress, cellular compensatory reactions can lead to an imbalance skewed toward injury as energy is consumed (126). Chronic stress, for example, increases the rate of DNA mutations in cells, causing dysfunction and even cell death (127), contributing to the development of many inflammatory diseases. Therefore, the immune effects induced by CORT may impact cell function through epigenetic modification. However, the specific genetic mechanisms require further exploration, such as studying the regulation of transcription, translation, and post-translational modification of different inflammatory genes by CORT under varying levels and durations of stress through omics studies. Additionally, exploring the influence of CORT on the DDR, such as the TCGA/DDR signaling pathway, is necessary for a deeper understanding.

## 3 Interaction pathway between corticosterone and immunity

### 3.1 The endocannabinoid system

The Endocannabinoid (eCB) system is widely present in various cell types and plays an essential role in metabolic signal transduction (128–130). It comprises (1) endogenous lipid transmitters such as endocannabinoids, including anandamide (AEA) and 2-arachidonoylglycerol (2-AG), (2) cannabinoid receptors (CBR), including type 1 (CB1R) and type 2 (CB2R), and (3) related enzymes such as N-acyl phosphatidylethanolamine-hydrolysis phospholipase D (NAPE-PLD), diacylglycerol lipase (DAGL), and degrading enzymes such as fatty acid amide hydrolase (FAAH) and monoacylglycerol lipase (MAGL) (131). The eCB system plays a crucial role in coping with stress. This is primarily reflected in the response and control of the eCB to CORT (end product of HPA axis), as well as the response and regulation of the CBR to CORT. As a marker of stress signals, CORT activates the eCB system (128).

Firstly, it is reflected in the mutual influence between CORT and eCB (2-AG and AEA). There are two situations here, namely short-term stress and long-term stress. The core difference between the two lies in whether the negative feedback function of the HPA axis (sensitivity) is normal. Danan D’s study (132) showed that an increase in CORT under short-term stress (1-2 hours after PSS, PSS is predator scent stress) stimulates compensatory responses of eCB, i.e., promotes the expression level of 2-AG (in cerebrospinal fluid). However, there was no significant difference in AEA levels (in cerebrospinal fluid). This study suggests that the response of eCB to acute stress is mainly through 2-AG. Similarly, Balsevich G’s study (133) found a positive correlation between CORT and 2-AG (elevation) in the short-term stress model, rather than AEA. Bedse G’s study (134) also confirmed this in the amygdala. Roberts CJ’s study (135) simulated short-term stress stimuli through forced swimming. The results also showed a positive correlation (elevation) between 2-AG levels and CORT in the hippocampus, amygdala, and prefrontal cortex (PFC). Morena M’s study (136) also found that under pressure (temperature stimulation), both 2-AG and CORT increased simultaneously, while AEA levels did not show significant changes. This indicates that the eCB system is mainly responsive to CORT by 2-AG under short-term stress. 2-AG participates in rapid and robust responses of stress regulation and promotes negative feedback function of the HPA axis (137, 138). Furthermore, it is widely believed that AEA is a regulatory molecule under the chronic action of GC, involved in downstream secondary signaling mechanisms of glucocorticoid receptor (GR) activation (139). Under short-term stress, based on negative feedback regulation of the HPA axis, these reactions will be self-downregulated afterwards (140), thereby restoring the homeostasis of the CORT and eCB systems.

Under long-term stress, the balance of HPA axis negative feedback is disrupted, and the compensatory response of eCB subsequently fails. At this point, the pathological effect of CORT dominates, which will disrupt the role of eCB (AEA and 2-AG). For AEA, Danan D’s study (132) showed that AEA levels (in hippocampus) were significantly reduced under long-term stress (1 week after PSS). Zada W’s study (141) showed that upregulating AEA expression through drugs (FAAH inhibitors) helps to reduce CORT levels and depressive behavior in a depression model (high CORT levels). Hill M.N’s research (139) has the same conclusion. Satta V’s study (142) simulated a chronic stress model by changing diet (stress duration of 5 weeks). The results showed that there were synergistic changes with the increase of CORT is, a decrease in AEA levels was observed in the amygdala, hippocampus, and caudate putamen. However, no significant changes in AEA were observed in the hypothalamus, nucleus accumbens, and PFC. Gray JM’s study (143) showed that under the action of CORT (capsule), AEA levels were reduced in both the PFC and amygdala. It can be seen that the increase of CORT under chronic stress will inhibit the expression of AEA. And AEA is considered the main type of eCB that responds to the chronic effects of CORT (144). However, under this condition (chronic stress), 2-AG is slightly controversial. Satta V’s study (142) showed that under chronic stress (dietary changes lasting up to 5 weeks), 2-AG significantly increased in the hippocampus, while there were no significant changes in the amygdala, caudate nucleus, nucleus accumbens, hypothalamus, and PFC. Gray JM’s study (143) showed that under the action of CORT (capsule), 2-AG was observed to increase in the PFC, while there was no significant change in the amygdala. On the contrary, recent studies by Danan D (132) have shown that under prolonged stress (1 week after continuous PSS), the levels of 2-AG in the hippocampus and hypothalamus are significantly reduced. From this perspective, there are more complex regulatory mechanisms under chronic stress. Moreover, the eCB system can accept crosstalk and feedback from various upstream and downstream signals, which makes its expression more confusing. For instance, distinct brain regions exhibit unique regulatory mechanisms. There are different signal transmission directions (compensatory and decompensated) in different response stages. And under the influence of diverse stressors, different dominant signals emerge, even though an elevation in CORT levels is consistently observed across all conditions. In more recently studies (145, 146), it is generally believed that the concentration of 2-AG increases under chronic stress. The increase of 2-AG under chronic stress is related to the inhibition of AEA and decreased sensitivity of CB1R (147, 148). Both 2-AG and AEA are constrained by the release mode of GC (such as CORT), and when CORT increases, both 2-AG and AEA change in opposite directions (143). However, a more detailed mechanism has not yet been fully determined.

Additionally, it should be noted that CORT regulates the eCB system not only by targeting 2-AG or AEA levels, but also by acting on CBR. CB activity mediated by CBR is a primary factor in maintaining the feedback regulation ability of the HPA axis (149, 150). Studies have shown that, pharmacological blockade or decreased expression and function of CB1R can disrupt the negative feedback of the HPA axis, leading to increased circulating CORT levels (151). Skupio et al. (152), CORT induces neuronal damage by activating CB1R on the mitochondrial membrane (mtCB1R), and this mechanism has different damaging effects in different brain regions. In mice, impairment of New Object Recognition (NOR) consolidation memory was induced in norepinephrine (NE) neurons of the locus coeruleus (LC), while in the hippocampus (HIP), impairment of NOR extraction memory was induced in GABAergic interneurons. In this pathway, it was observed that CORT led to an increase in 2-arachidonoylglycerol (2-AG) levels. These pieces of evidence suggest that the eCB system is a vital component in the response to stress. Furthermore, downstream of the eCB system, it is intricately linked to the immune system, serving as a crucial bridge for the interaction between CORT and the immune response (153, 154).

**Figure 4:**
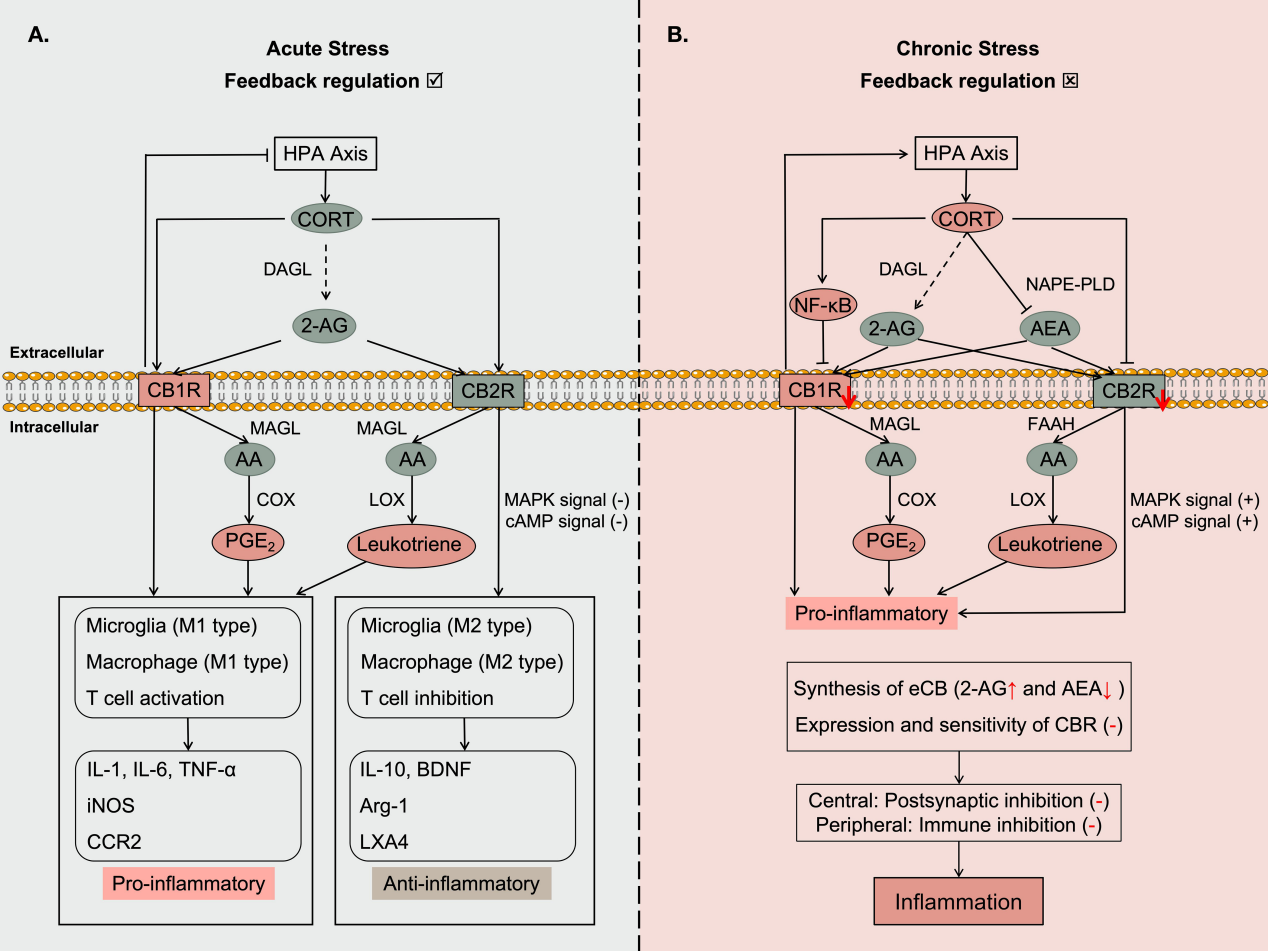
The immune effect of CORT through the eCB system. **(A)** Under acute stress, 2-AG is the main type of eCB that responds to stress (CORT). Mediate the inflammatory response via CB1R, while inhibiting inflammation through CB2R. This achieves immune balance. And ultimately end the stress response through negative feedback regulation of the HPA axis. **(B)** Under chronic stress, CORT levels remain elevated, leading to the continuous activation of both the eCB system and the immune system. In this context, the low expression of AEA reflects the depletion of the eCB system, while the high expression of 2-AG represents the body’s attempt to counteract the effects of elevated CORT. As a sustained stress signal, CORT promotes the dominance of CB1R while CB2R is inhibited. This shift drives the immune response toward a pro-inflammatory state. Eventually, the eCB system becomes exhausted, rendering it incapable of effectively inhibiting inflammation. The straight arrow represents the direct promoting effect. The dashed arrow represents the indirect promoting effect. The minus line represents the inhibitory effect. The plus sign "(+)" represents signal enhancement. The minus sign "(-)" represents a weakened signal. The red down arrow represents a decrease in the expression level of the molecule. The red up arrow represents an increase in the expression level of the molecule.

The signal transduction of the CB system to the immune system involves multiple pathways, including direct communication and indirect communication through arachidonic acid signaling. This regulates the function of immune cells, such as proliferation, secretion, and apoptosis (155, 156). The realization of immune activation and immune suppression mainly depends on the dual channels of the CB system, involving two G protein-coupled receptors, CB1R and CB2R, and the activation and sensitivity of these receptors. CBR mainly functions in central neurons. Among them, CB1R is primarily expressed in microglia, neuronal endings, and astrocytes, whereas CB2R is mainly expressed in microglia and glial cells (157, 158). Currently, it is believed that CB1R is primarily associated with promoting the production of inflammatory mediators, while CB2R is mainly involved in inhibiting inflammation, thereby communicating with immune cells (154). And, microglia are essential immune cells in the central nervous system as they possess significant neuroimmune regulatory abilities. Therefore, in neuroimmunity, the immune regulation of eCB cannot be separated from the immune function of microglia. They exhibit two activation states: classical activation (M1 polarization) and alternative activation (M2 polarization) (159). M1 polarization is associated with pro-inflammatory effects. In contrast, M2 polarization has anti-inflammatory and neurotrophic properties (160, 161). As shown in **Figure 4**.

#### 3.1.1 Direct communication between CORT and immunity

About direct communication, research has found that in M1-type microglia, the binding of 2-AG to CB1R increases pro-inflammatory mediators. However, in M2-type microglia, the binding of 2-AG to CB2R enhances the expression of the anti-inflammatory cytokine IL-10 and the solubilizing factor lipoxin-4 (LXA4) (162). LXA4 can induce apoptosis of inflammatory cells and participate in immune suppression (163). Additionally, activation of CB2R facilitates the transition of microglia from M1 type to M2 type (164), leading to a decrease in the expression of iNOS, a marker of M1 activity, and an increase in the expression of Arg-1, a marker of M2 activity (160). Administration of exogenous 2-AG in inflammatory model mice promotes an increase in the number of M2-type microglia (162). CB2R signaling inhibits the expression of pro-inflammatory mediators iNOS and CCR2 in IFN-γ-induced inflammatory mouse microglia (165). CCR2 is a chemokine receptor associated with immune cell recruitment, reflecting that the immune regulation by 2-AG depends on the activation status of microglia and the sensitivity of corresponding CBRs in this state. Other studies have shown that AEA and 2-AG inhibit T cell proliferation and reduce IL-1, IL-6, IL-9, IL-17 and TNF-α levels by activating CB2R (166). CB2R can simultaneously inhibit adenylate cyclase (AC) activity, thereby inhibiting the cyclic adenosine monophosphate (cAMP) signaling pathway and lymphocyte activation. This demonstrates that the CB system regulates immune cell and cytokine secretion by activating different CBRs and maintains homeostasis of the internal environment through a dual effect of pro-inflammatory and anti-inflammatory actions (167). During this process, inflammation results from the interaction between pro-inflammatory and anti-inflammatory substances.

#### 3.1.2 Indirect communication between CORT and immunity

Indirect communication with the immune system is accomplished through the transmission of arachidonic acid-like signals (168). The biosynthesis of arachidonic acid (AA) involves the oxidation of polyunsaturated fatty acids by cyclooxygenase (COX) and lipoxygenase (LOX). Interestingly, endocannabinoids (AEA and 2-AG) are derivatives of AA and are influenced by the same oxidative metabolic pathway. Prostaglandins (PG), such as PGE2 (via the COX pathway) and leukotrienes (via the LOX pathway), are the main metabolic products (169). While endocannabinoids exert their anti-inflammatory effects through CB2R, they are degraded into AA by FAAH and MAGL, and enter the AA synthesis pathway to produce inflammatory mediators PGE2 and leukotrienes. These processes rely on the production of nitric oxide (NO) to provide pro-inflammatory effects and enhance immune responses (170). In the LPS-induced inflammatory mouse model, inhibiting MAGL activity reduces the secretion of PGD2, PGE2, PGF2α, and pro-inflammatory cytokines IL-1α, IL-1β, IL-6, and TNF-α in the brain (171). This phenomenon is also observed when inhibiting COX-2 (172). Thus, activating the immune system includes both pro-inflammatory and anti-inflammatory effects. This process requires significant energy and substrate consumption, as well as the continuous operation of organelles. The final result depends on which signaling pathway is predominantly and continuously activated. Persistent inflammation and cell damage are the outcomes of the sustained action of stress hormones.

Conversely, immune cells can coordinate CB signaling by regulating the transcription, synthesis, uptake, and degradation of CB components. Studies have shown that CB1R expression and AEA levels in lymphocytes are reduced following intervention with the anti-inflammatory cytokine IFN-β (173). LPS-induced activation of mouse macrophages results in an increase in platelet-activating factor (PAF), which promotes the synthesis of AEA and 2-AG due to decreased expression of FAAH (174). The study conducted by Standoli S (175) reveals that inhibiting FAAH and activating the CB2R can effectively prevent the production of TNF-α and IL-1β induced by LPS. Additionally, immune cells can directly participate in the degradation of AEA and 2-AG (dependent on concentration feedback) to terminate CB signaling (176, 177). Other studies have demonstrated that in the LPS-induced inflammatory mouse model, CB1R expression decreases at the membrane protein level while CB1R mRNA expression increases (178), which may represent an adaptive regulation of the body’s response to inflammation, primarily manifested at the protein level.

### 3.2 TREM2 mediated immune regulation

Triggering receptor expressed on myeloid cells-2 (TREM2) is a transmembrane receptor of the immunoglobulin superfamily expressed in various immune cells such as DCs, microglia, and macrophages (179–181). Upon binding to its ligand, TREM2 interacts with DNAX-activating protein of 12 kDa (DAP12) to induce phagocytosis of tissue fragments and promote anti-inflammatory properties (182). This interaction is related to downstream signaling pathways involving PLCγ2, PI3K, and AKT (183–185). DAP12 is also a transmembrane receptor widely present on the surface of immune cell membranes. In microglia, TREM2 is responsible for synaptic inhibition and establishing normal brain connections, maintaining innate immune homeostasis and cellular metabolism (186). TREM2 also participates in the M1/M2 polarization of microglia to regulate inflammatory responses. Upregulation of TREM2 expression promotes the transition of microglia from M1 to M2 type, enhancing their phagocytic function, reducing the release of inflammatory mediators, and inhibiting the inflammatory cascade response. Conversely, downregulation of TREM2 expression promotes inflammation (187).

Recent studies have shown that CORT affects the immune function of microglia through TREM2, inducing the production of inflammatory factors (188, 189). Cell experiments have demonstrated that when CORT concentration exceeds 1 μM, it significantly inhibits the proliferation of microglia (BV2), with the degree of inhibition becoming more pronounced at higher CORT concentrations, almost completely inhibiting growth at 500 μM CORT. Intervention using 10 μM CORT was found to significantly decrease TREM2 expression. The experiment also revealed an upregulation in the expression of M1-type markers, including iNOS and CD16, in microglia, while the expression of M2 biomarkers CD206 and Arg-1 declined. Additionally, there was an increase in the levels of pro-inflammatory factors such as TNF-α, IL-1β, and IL-6, coupled with a decrease in the anti-inflammatory factor IL-10, which are in line with previous research (187). Transfecting TREM2 into the cells was found to reverse this phenomenon, while knocking out TREM2 in mice increased the levels of TNF-α, IL-1β and IL-6 and decreased the levels of IL-10. These results indicate that inhibiting TREM2 is one of the mechanisms by which CORT mediates the pro-inflammatory effects of microglia.

The JAK2/STAT3 signaling pathway is involved downstream of TREM2 in the immune regulation of CORT. This pathway plays an important role in the development of innate and acquired immune cells, activation of IFN, and expression of inflammatory cytokines (190, 191). It is a significant mediator in synaptic transmission, where enhanced synaptic transmission activates the JAK2/STAT3 signaling pathway to promote the production of inflammatory factors (192). Studies have shown that chronic unpredictable mild stimulation (CUMS) induces depression-like behavior and the release of inflammatory factors in rats by activating the IL-6/JAK2/STAT3 pathway in the hypothalamus (193). Further research has indicated that this process is related to CORT inhibiting TREM2 expression. Overexpression of TREM2 can reverse this phenomenon to promote the transformation of microglia from M1 type to M2 type (189), thereby exerting anti-inflammatory effects.

Regulating downstream signaling pathways of inflammation through TREM2 involves not only JAK2/STAT3 but also NF-κB and PI3K/AKT pathways, which are implicated in NLRP3 inflammasome-mediated neuroinflammation (194). During stress, CORT recognizes peripheral signals to activate the intracellular pattern recognition receptor (PRR) NLRP3. Subsequently, ASC binds to pro-caspase-1 to form activated caspase-1, which promotes the maturation of IL-1β and IL-18, thereby exerting pro-inflammatory effects (195). Therefore, the expression of NLRP3 and activated caspase-1 are key markers of inflammation. TREM2 is widely recognized as a key protein molecule that inhibits the inflammatory cascade response (196). Recent studies (187) have shown that overexpression of TREM2 effectively reduces the expression levels of NLRP3 and pro-caspase-1 proteins in rats, as well as the secretion of the inflammatory mediators IL-1β and IL-18, both *in vivo* and *in vitro*. This anti-inflammatory effect is associated with the inhibition of the TLR4/MyD88/NF-κB signaling pathway and the upregulation of PI3K/AKT phosphorylation levels (197, 198). These findings are consistent with previous results. The upregulation of TREM2 promotes M2 polarization of microglia and reduces the secretion of inflammatory mediators, thereby exerting neuroprotective effects. Inhibiting the NF-κB signaling pathway and activating the PI3K/AKT signaling pathway are essential for these effects (199–201).

**Figure 5:**
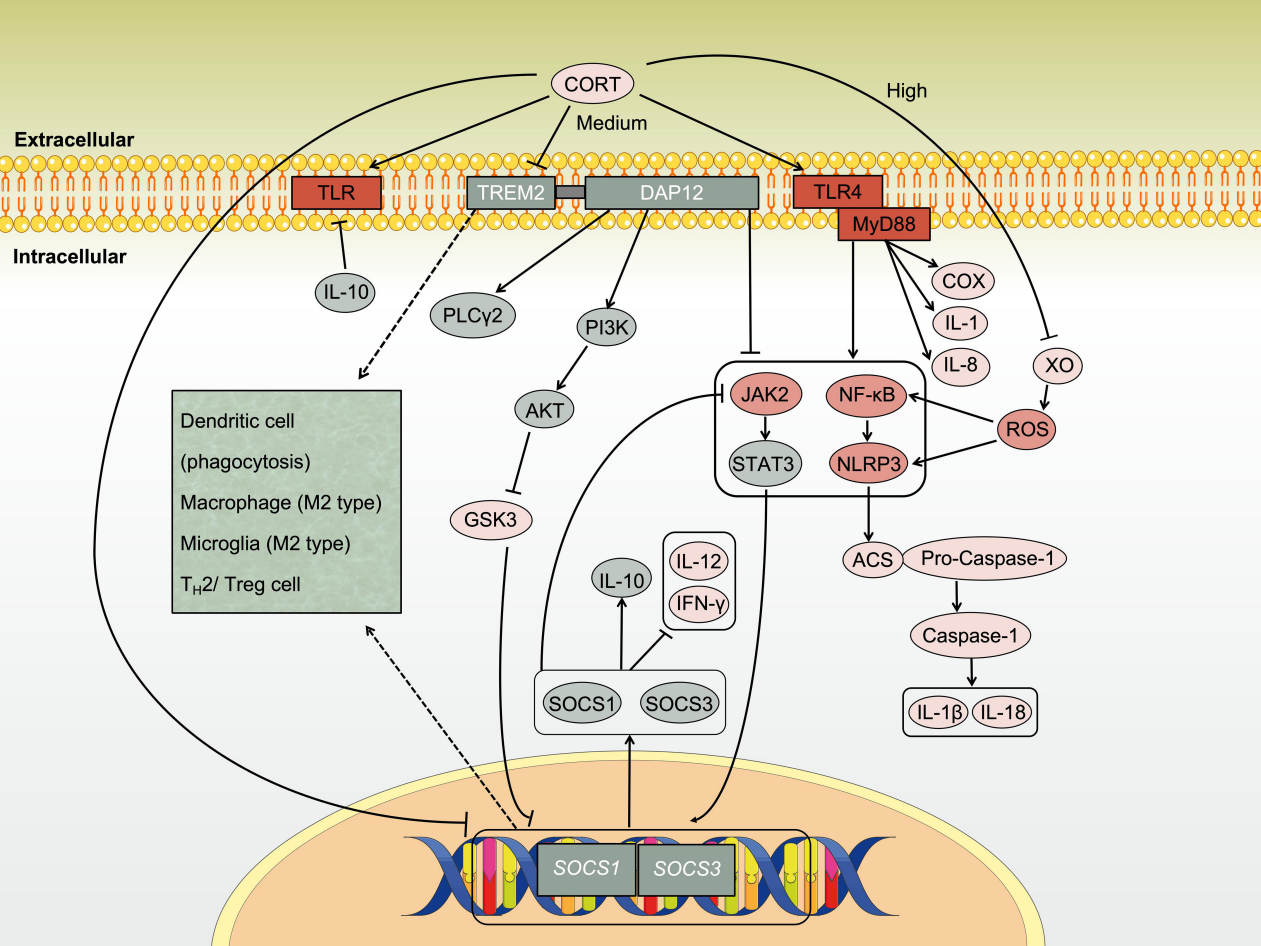
Regulation of immune activity by CORT through TREM2. Under chronic stress, CORT stimulates inflammatory signaling through Toll-like receptors (TLRs), which involving key pathways such as NF-κB, NLRP3 inflammasome, and JAK/STAT, which collectively drive the progression of inflammation. TREM2 is a crucial membrane receptor protein with immunomodulatory functions that help inhibit inflammation. However, sustained high levels of CORT disrupt the ability of immune cells to suppress inflammation by inhibiting the TREM2/PI3K/AKT signaling pathway.

The effects of CORT on downstream signaling pathways are concentration-dependent, with both upregulating and downregulating impacts on the same pathway, thereby exerting pro-inflammatory or anti-inflammatory properties. Wu et al. (202) demonstrated a dual effect of different CORT concentrations on an LPS-induced mouse macrophage inflammation model. When the concentration of CORT was below 300 ng/ml, the protein expression level of NLRP3 in mouse macrophages was significantly upregulated. However, when the concentration of CORT exceeds 300 ng/ml, the protein expression level of NLRP3 gradually decreases, reaching its lowest level at 700 ng/ml, along with a decrease in activated caspase-1 expression. Xanthine oxidase (XO) primarily mediates the production of mitochondrial reactive oxygen species (ROS) (203), which may be responsible for activating NLRP3 (204, 205). Research has found that CORT regulates the pro-inflammatory factor NLRP3 through the enzyme activity of XO (202). Higher concentrations of CORT (700 ng/ml) downregulate the mRNA and protein expression of NLRP3 by inhibiting the activity of XO, thereby modulating the body’s inflammatory response. Thus, while the signaling pathways affected by CORT may be consistent, the specific role of immune promotion or immune suppression depends on the circulating concentration, as illustrated in **Figure 5**.

### 3.3 SOCS1 and SOCS3 mediated immune regulation related to CORT

Suppressor of Cytokine Signaling 1 (SOCS1) is a negative regulatory factor that effectively prevents the overactivation of the immune system (206), and its transcription is regulated by the JAK/STAT signaling pathway. Additionally, SOCS1 can bind to the catalytic site of JAK2 through its specific enzyme activity inhibitory region, thereby inhibiting JAK2/STAT3 signal transduction (207). Inhibiting SOCS1 has also been found to promote the proliferation of CD4^+^ and CD8^+^ T cells (208). Studies have observed that CORT reduces the expression of SOCS1 in microglia, thereby promoting the expression of pro-inflammatory factors TNF-α, IL-1β, and IL-6 (189). These factors promote the polarization of microglia towards the M1 type. Subsequently, activated microglia exacerbate synaptic damage by releasing pro-inflammatory factors, promoting the accumulation of phosphorylated tau, and inducing neuronal apoptosis (209), indicating that the activation of microglial immune function by pro-inflammatory concentrations of CORT is achieved by inhibiting SOCS1. However, CORT can also activate the JAK2/STAT3 signaling pathway, suggesting that there is another mechanism by which CORT inhibits SOCS1 that warrants further exploration.

SOCS3 is an IL-10 inducible gene, and IL-10 primarily achieves immunosuppressive effects by inactivating myeloid cells and inhibiting the production of inflammatory factors (210). IL-10 typically induces STAT3 activation, which inhibits TLR-mediated pro-inflammatory cytokine expression at the transcriptional level. Furthermore, IL-10 induces the polarization of microglia towards the M2 type (211). The expression of IL-10 is not entirely dependent on the PI3K/AKT pathway, and the IL-10-induced *SOCS3* gene is not regulated by it. However, the expression of IL-10 induced by other genes, such as *ARNT2* and *Autotaxin*, depends on the PI3K/AKT pathway (212). Downstream of p-AKT, IL-10-induced gene expression is further increased by inhibiting GSK3 activity (mainly GSK3α, followed by GSK3β). Although it has been found that cAMP response-element protein (CREB) is one of the targets of GSK3, it has also been shown that CREB is not involved in GSK3 regulation of signal transduction between IL-10 (213). Downstream of IL-10, the PI3K/AKT pathway is involved in IL-10 inhibition of TLR-induced synthesis of COX2, IL-1 and IL-8, but not in IL-10 inhibition of TNF-α synthesis (212). This indicates that the PI3K/AKT pathway selectively regulates the immune response of IL-10. Upstream of the PI3K/AKT pathway, CORT inhibits the PI3K/AKT pathway, while TREM2 promotes it. In summary, the specific regulatory mechanism is shown in **Figure 5**.

### 3.4 Programmed cell death

#### 3.4.1 FOXO3a and ROS

CORT can activate immune cells and release inflammatory mediators to respond to the immune environment through various signaling pathways. However, alongside adaptation and coping, damage also occurs. This is especially evident in chronic stress, where repair and regulation are less effective than injury response. Under the cytotoxic effect of CORT, signals of abnormal intracellular metabolism continuously drive immune regulation and, over time, initiate autonomous cell death (214). Chang et al. (215) showed that CORT (100 μM)-induced neuronal apoptosis results from a combination of multiple pathways, including the mitogen-activated protein kinase (MAPK) cascade reaction (MAPK/ERK signaling pathway and p38 MAPK signaling pathway) and the PI3K/AKT/FOXO3a signaling pathway. These intracellular kinase signaling cascades are believed to be responsible for promoting neuronal survival (215, 216). The MAPK/ERK signaling pathway is mainly responsible for regulating cell viability and proliferation (217). The p38 MAPK signaling pathway primarily regulates cell differentiation, antioxidant stress survival, inflammation, and the cell cycle (218, 219). Research has shown that high concentrations of CORT increase intracellular ROS and FOXO3a nuclear accumulation by inhibiting these signaling pathways based on the observed decreased phosphorylation of p38, ERK, PI3K, and AKT. This inhibition leads to an increased rate of cell apoptosis (215). FOXO3a is a transcription factor that triggers cell apoptosis, characterized by a forkhead domain that binds to DNA, thus directly participating in gene transcription (220). However, FOXO3a within the nucleus is limited. Part of FOXO3a is phosphorylated and translocated from the nucleus to the cytoplasm, where it regulates important physiological processes such as energy metabolism, cell apoptosis, and oxidative stress and is ultimately degraded, preventing cell apoptosis (221, 222). CORT reduces FOXO3a phosphorylation by inhibiting the PI3K/Akt signaling pathway, causing its accumulation in the nucleus and inducing cell apoptosis (215, 223).

The production of ROS plays a crucial role in stimulating the continuous activation of immune cells under sustained stress (224) as ROS act to recruit more immune cells, prompting them to produce pro-inflammatory factors (225). However, the damaging effects of ROS cannot be overlooked. Intracellular ROS induce the activation of transcription factors such as NF-κB and MYC, which in turn synthesize both pro-apoptotic and anti-apoptotic factors, thereby initiating apoptosis programs (226, 227). Pro-apoptotic genes, including *Apaf1* and members of the Bcl-2 family like *Bad*, *Bbc3*, *Bik*, and *Pmaip1*, are upregulated. Furthermore, downstream molecules such as caspase-3 and caspase-6 are activated in T cells. Interestingly, the use of GR antagonists, which block the effect of CORT, can reverse apoptosis (102). Previous studies have demonstrated that stress induces the maturation and apoptosis of CD4 and CD8 T cells, leading to the depletion of the T cell pool (228). Prolonged exposure to antigens can drive T cells into a state of depletion, where immature T cells become the primary force of immunity. However, due to insufficient energy and abnormal cellular metabolism, overall immune function shifts towards an immunosuppressive state. This alteration affects downstream signaling cascade reactions and epigenetic processes (71, 229). For instance, lactate dehydrogenase A (LDHA) plays a role in providing energy for T cell activation and proliferation by participating in lactate metabolism, exhibiting non-classical enzyme activity, and regulating oxidative stress responses (230). However, when ROS synthesis surpasses decomposition, leading to cytoplasmic accumulation, LDHA function is inhibited (231). In response to stress, cells adjust the intensity of multiple gene expressions, triggering intracellular cascade reactions that may lead to exhaustion and, ultimately, cell death (71).

#### 3.4.2 GSDMD and NLRP3

In the central nervous system, huang et al. (198) reported that CORT promotes the expression of key apoptotic proteins GSDMD and GSDMD-N in microglia. Thus, microglia not only produce inflammatory mediators that enter the bloodstream through pro-inflammatory signaling by CORT but also activate their apoptotic pathways, leading to programmed cell death after sustained immune activity. This process consumes significant energy and substances, and the inflammatory mediators entering the circulation act as new signaling molecules, inducing further inflammation throughout the body. If left untreated, this can cause inflammatory damage and impair tissue function. Peripherally, chronic stress-induced elevated CORT causes macrophage infiltration in the spleen of mice. It has been observed that as the phagocytic function of macrophages weakens, pyroptosis increases, and autoantibody production decreases, resulting in immunosuppressive effects (232, 233). This pathway relies on the activation of NLRP3 inflammasomes rather than the P-selectin pathway (232) and corresponds to the previously mentioned mechanism but with slightly different outcomes. Research has found that under the influence of CORT, the expression of NLRP3 and caspase-1 in macrophages increases (caspase-1 promotes IL-1β maturation), leading to an increase in the circulating pro-inflammatory cytokine IL-1β (232). NLRP3 inflammasomes, including NLRP3 and caspase-1, have pro-apoptotic and inflammatory effects (234). Using NLRP3 inhibitors (OLT1177) and caspase-1 inhibitors (Z-WEHD-FMK) can block the apoptotic pathway and subsequent cascade events of inflammation (232, 235), indicating that the pro-inflammatory effect continues to erupt within immune cells, eventually ending immune activity through cell death.

#### 3.4.3 Notch signaling pathway

After chronic stress triggers an increase in circulating CORT levels, it also activates the Notch signaling pathway, inducing immune suppression and splenocyte apoptosis (233). Activation of the Notch signaling pathway is observed with increased expression of NICD1, DLL1, DLL4, Jagged 2 and Hes1, while the expression of DLL3, Jagged 1 and Hes5 remains unchanged. Concurrently, decreased IFN-γ levels and increased IL-4, caspase-8, and caspase-3 levels are noted. Song et al. also demonstrated that chronic stress-induced splenic apoptosis is mediated through the death receptor pathway (236). Additionally, TLR4 activation has been found to be implicated in immune suppression induced by increased CORT under stress (237). These findings suggest that both immune activation and immune suppression are closely related to inflammation. It is important to note that immunosuppressive characterization may result from immune overactivation, where anti-inflammatory signaling pathways are less dominant compared to pro-inflammatory pathways.

#### 3.4.4 miR-155

In addition, the increase in CORT caused by chronic stress downregulates the expression of miR-155, resulting in decreased BCL-6 levels and increased FBXO11 levels. This impairs the germinal center response of B lymphocytes and the production of IgG1 antibodies, thereby inhibiting immune function (238). The germinal center is a histological structure formed during the maturation and differentiation of B cells into plasma cells and memory B cells. BCL-6 is a transcription factor essential for the formation of germinal centers (239, 240). The SKP1-CUL1-Fbox protein (SCF) ubiquitin ligase complex containing FBXO11 induces ubiquitination and degradation of BCL-6. Excessively high levels of FBXO11 hinder B cell differentiation and induce B cell apoptosis, while low levels promote lymphatic proliferation and carcinogenesis (241, 242). The balance between FBXO11 and BCL-6 levels is essential for B cells to maintain normal immune function. Apoptosis, widely regarded as programmed cell death activated by highly inflammatory and cytotoxic metabolites (243, 244), is associated with stress-induced elevated CORT (245). These pathways mediate the activation and damage of immune cells by CORT. The inflammatory factors released during injury re-enter the bloodstream, reactivate the immune system, and attack vulnerable areas of the body by identifying abnormal signals and generating signal transmission to trigger new inflammatory reactions.

#### 3.4.5 TFEB

Transcription factor EB (TFEB) belongs to the MiT/TFE family of basic helix-loop-helix leucine zipper transcription factors and serves as a pivotal regulator of autophagy and lysosomal biogenesis (246). Additionally, TFEB has been implicated in governing energy homeostasis and cellular responses to various stressors, such as nutrient deprivation, endoplasmic reticulum stress (ERS), mitochondrial autophagy, and pathogen invasion (247, 248). It is involved in multiple signaling pathways, including mTORC1, Wnt, and AKT pathways (249). Phosphorylation of TFEB at the S401 site facilitates redox reactions and the release of growth factors to adapt to stress conditions (250). Recently, TFEB has emerged as a key player in controlling inflammatory responses by inhibiting pro-inflammatory cytokines and modulating immune cell differentiation (251, 252). The inhibition of TFEB has been implicated in promoting immune evasion (253). Recent studies suggest that this adaptive regulation can be inhibited by p38 MAPK or blocked by substrate depletion (250). The p38 MAPK/TFEB signaling axis suppresses the expression of multiple immune-related genes in monocytes, as well as cytokines (such as IL-1β and LIF), chemokines (including CXCL1, CXCL3, CXCL8, and CCL5), and crucial immunomodulators (such as IFNGR2 and EREG). Consequently, this leads to aberrant macrophage differentiation and impaired polarization. Enhanced nuclear translocation of TFEB boosts the expression of lysosomal proteins and superoxide dismutase (SOD), ultimately diminishing ROS levels and suppressing ferroptosis, thus exerting a protective effect (254). These findings indicate the pivotal role of TFEB in immune and redox regulation, suggesting potential avenues for further exploration into its regulatory mechanisms.

#### 3.4.6 TAM family of receptor tyrosine kinases

The increase in CORT induced by stress also activates the GR-MERTK signaling pathway in astrocytes, leading to heightened phagocytosis of excitatory synapses by astrocytes in cortical regions, thereby eliciting depressive behavior in mice (255). MERTK belongs to the TAM family, which encompasses TYRO3, AXL, and MERTK, and is comprised of RTK. This family acts as a bridge between its structurally homologous ligands, GAS6 and PROS1, and binds to phosphatidylserine on the apoptotic cell membrane (PtdSer) to mediate immune regulation (256).

**Figure 6:**
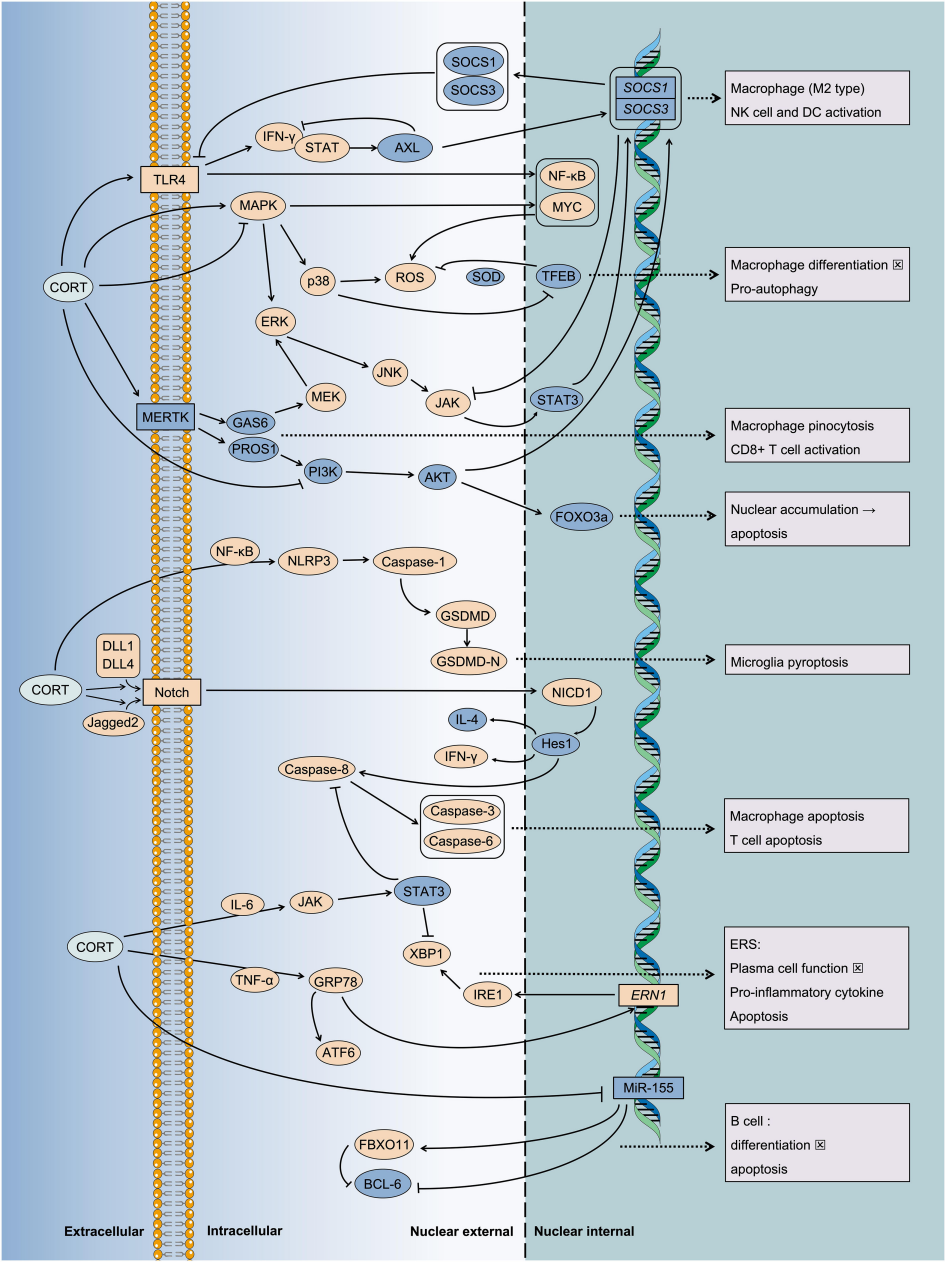
Multiple pathways mediate the inflammatory injury of CORT induced cell death. Under chronic stress, the sustained action of CORT leads to programmed cell death. This phenomenon indicates an immune imbalance, characterized by the ongoing progression of inflammation. It is associated with the transmission of numerous intracellular stress signals and the subsequent regulation of gene expression. For instance, key signaling pathways such as MAPK, JAK/STAT, Notch, and NF-κB, etc. And some key regulatory proteins such as MERTK, TFEB, etc.

Recently, TAM receptors have received significant attention as potential therapeutic targets for their ability to control inflammation and immunosuppression. Present research reveals that TAM receptor activation can inhibit immune activity downstream through various pathways, including MEK/ERK, PI3K/AKT, and JAK/STAT pathways (257). For instance, in macrophages, MERTK governs its phagocytic function (258), while AXL signaling promotes a shift towards the M2 phenotype in macrophages, resulting in increased expression of IL-10 and TGF-β, and decreased expression of IL-6, TNF-α, and G-CSF (259). Furthermore, TAM signaling inhibits the activation of NLRP3 inflammasomes in macrophages, thereby attenuating the inflammatory pathway and preventing chronic macrophage activation (260). Similar to NK cells, AXL signal transduction reduces their secretion of IFN-γ and diminishes their killing function (261). Similarly, in DCs, TLRs activation upregulates AXL expression through STAT1 signaling transduction. Subsequently, AXL inhibits IFNAR-STAT1 signaling and induces the expression of SOCS1 and SOCS3, thereby negatively regulating TLR signal transduction, inhibiting the inflammatory response and terminating DC activation of T cells (262). Activated T cells secrete protein S (PROS1) as an additional feedback mechanism for DCs to assist in TAM signaling to suppress immune responses (263). Conversely, activated T cells increase MERTK expression and activate MERTK signal transduction, a co-stimulatory pathway that promotes CD8^+^ T cell activation (263, 264). Inhibiting MERTK signal transduction reduces IFN-γ secretion and CD8^+^ T cell proliferation (263). Furthermore, studies have revealed co-expression of MERTK and PD-1 in activated T cells (265–267). The opposing regulatory effects of TAM receptors on innate immune cells and T cells, as well as the dual effects of MERTK, have roused the interest of researchers. Currently, more mechanisms for regulating TAM receptors are being investigated. From this, it can be evident that CORT induces programmed cell death through multiple signals, thereby impairing immune function and promoting inflammation progression. In summary, the specific mechanism is shown in **Figure 6**.

#### 3.4.7 Cell competition

It is also worth noting that a state closely related to cell apoptosis is inter-tissue cell competition, which involves signal pathways correlated with GR signal transduction induced by CORT, warranting attention. Intercellular interaction in cell competition aims to maintain tissue health and cellular homeostasis (268). Due to its involvement in immune regulation across various diseases, it has become a research hotspot, including tumor immune escape and neurodegenerative diseases (269, 270). Cell competition operates on the principle of “survival of the fittest” to sustain tissue physiological function and internal environment homeostasis (269). Disruption in the balance of cell competition results in “winner” cells and “loser” cells, where loser cells experience slowed proliferation and incomplete apoptosis, while winner cells exhibit accelerated proliferation (271, 272). Research suggests that reasons for the failure of cell competition are associated with chronic activation of the TLR pathway (273), p53/DDR pathway (274), c-Jun N-terminal kinase (JNK) pathway, Janus kinase (JAK)/signal transducer and activator of transcription (STAT) pathways (275), and oxidative stress response pathways (231).

TLR pathway activation not only initiates inflammation but also induces cell apoptosis (276). The activation of the p53 pathway is related to DDR, with the related genes *Mre11*, *Lig3*, *Lig4*, and *Ku80* being upregulated in DDR and are considered targets of p53 (277, 278). The JNK pathway primarily participates in cell proliferation and death (279). Upon activation, JNK upregulates the expression of genes such as *TRE-dsRED*, *Scarface* and *Reaper*, which are involved in competition failure to induce cell apoptosis (280). Moreover, it can inhibit cell proliferation rate by impacting protein synthesis, potentially contributing to competitive failure (231, 281). However, the specific mechanism by which it inhibits cell proliferation rate remains unclear. The JAK/STAT pathway are primarily involved in cell proliferation, immune response, and inflammatory response (275). The state of “loser” cells is associated with JAK/STAT pathway activation, with its target genes *Socs36E* and *Chinmo* observed to be upregulated. This mechanism is activated by JNK signaling upstream, with unpaired ligand 3 (Upd3) increasing horizontally to enhance signal transduction (231). Due to interaction between competing parties, “loser” cells can promote the proliferation of “winner” cells in competition (relative to their own proliferation rate) (282). Subsequently, “loser” cells may undergo apoptosis. Interestingly, the mechanism by which “winner” cells accelerate proliferation also involves JAK/STAT signaling (231). This highlights the dual role of the JAK/STAT pathway controlled by Upd3, which promotes apoptosis in “loser” cells and accelerates proliferation in “winner” cells. Therefore, the subsequent effects of the JAK/STAT signaling pathway may synergize with other mechanisms and are related to the properties of its upstream ligands.

Oxidative stress response is one of the significant triggers for cell competition and subsequent failure (283). Upregulation of genes associated with the expression of glutathione (GSH), glutathione transferase (GST), and cytochrome P450 oxidases (CYP450) has been observed in potential “loser” cells, with most of these genes being targets of Nrf2 (231). Nrf2 is a transcription factor that responds to stress environments by upregulating genes related to antioxidant function. Activation of the Nrf2 pathway in cells (dependent on transcription factors IRBP18 and Xrp1) is associated with the “loser” state (284). However, knockdown or overexpression of Nrf2 accelerates cell death and renders cells more sensitive to becoming “losers” (231). Nrf2 also demonstrates a dual effect depending on concentration; “loser” cells triggered by oxidative stress response rely on adaptive regulation of the Nrf2 pathway to maintain cellular homeostasis, but excessive accumulation of Nrf2 in “loser” cells relies on JNK to induce cell death (285), highlighting the importance of balance in the process of cell development and normal function and illustrating that both excessive and insufficient responses can have detrimental effects.

Current experiments have shown that p53 and JNK need to collaborate with other mechanisms to induce competition failure, such as the JAK/STAT pathway and the Nrf2 pathway (282), which indicates that cells that fail in competition are determined by multiple factors working together. Under stress, the activation of various intracellular signaling pathways makes cells “sensitive,” and at the same time, they become “fragile” due to their easier triggering of death programs. Under the influence of adjacent cells, such as through cell-cell communication or competition for resources, individual cells will undergo processes that determine their fate, balancing between adaptive survival and apoptosis outcomes. However, it is currently unclear which pre-existing conditions make cells potential “losers” and trigger cell competition. Based on the series of signal transduction induced by CORT mentioned above, it is speculated that chronic stress-induced elevated CORT may be one of its inducing factors. However, more experimental evidence is still needed for validation.

### 3.5 Endoplasmic reticulum stress

The endoplasmic reticulum (ER) is a vital organelle within the cytoplasm, crucial for various intracellular processes such as protein folding, modification, and calcium storage (286). Its functionality is intertwined with energy metabolism and facilitates communication between cells by providing proteins for intracellular and extracellular signal transduction (287). Proper protein synthesis and processing rely on the ER’s normal function. ERS serves as an alert for aberrant ER function, initially aiming to adapt to changing environments and restore ER function patterns. This response involves several mechanisms: (1) inhibition of upstream transcription and translation programs, which reduces the influx of new proteins into the ER; (2) induction and enhancement of protein repair gene expression to reduce protein folding errors; and (3) Promote protein degradation function to remove misfolded proteins (288, 289). Once the adaptive mechanism is activated, if the stressor persists, it may gradually cause the ER function to deviate from normal, resulting in persistent ERS (290). Although the adapted program is the optimal solution under current conditions, if the intracellular stress signal persists and the ER function cannot return to normal, the apoptotic program might be initiated (291).

Unfolded protein response (UPR) is an important cellular mechanism in response to ERS (292). This response involves the transition of glucose regulatory protein 78 (GRP78) from a bound to a free state, leading to an increase in GRP78 levels. Subsequently, downstream transcription factors such as X-box binding protein 1 (XBP1) and activating transcription factor-6 (ATF6) are activated (293), initiating the transcription of genes involved in ERS-related responses. XBP1 and ATF6, as nuclear transcription factors induced by ERS, play pivotal roles in intercellular signaling and can modulate downstream cellular functions (294–296). Research indicates that XBP1 and ATF6 not only stimulate the transcription of ER membrane and calcium reticulum protein genes during ERS (297), but also contribute to the generation of certain inflammatory mediators (298). Furthermore, XBP1 is essential for the production and secretion of antibodies by plasma cells (299). ERS-induced alterations in intracellular calcium homeostasis and protein quantity and structure represent adaptive immune responses to stress (300).

ERS within immune cells can significantly influence various immune functions, including antigen presentation (289), plasma cell differentiation, antibody production (300, 301), and T cell response to antigens (302). These alterations can significantly impact the onset and progression of inflammation, which is a key contributor to various tissue diseases. Among immune cells, macrophages are key in producing pro-inflammatory factors and orchestrating immune responses. Zhou et al. (303) demonstrated that low concentrations of CORT at 10 and 50 ng/ml induced ERS in macrophages, leading to notable increases in glucose regulatory protein 78 [GRP78; an important regulatory protein in the ERS process (304)] expression at both mRNA and protein levels. Furthermore, only 50 ng/ml of CORT has been shown to increase XBP1 expression, while no significant change was observed in activating transcription factor-6 (ATF6) levels. Evaluation of macrophage immune activation through adhesion index, chemotaxis index, and tumor necrosis factor-alpha (TNF-α) production revealed that CORT induces ERS and enhances immune function via GR activation on macrophages. The maximal immunostimulatory effect of CORT was observed at a concentration of 50 ng/ml, while concentrations of 100 ng/ml, 500 ng/ml, and 1000 ng/ml showed no such effect. Dhabhar et al. (305) further suggested that 50 ng/ml of CORT roughly corresponds to the physiological levels produced by the body during stress and is sufficient to exert immune-stimulating effects on macrophages. These results show the role of chronically elevated CORT levels in continuously triggering immune and inflammatory responses until normal cellular function is compromised.

ERS represents one of the pathways through which chronic stress induces apoptosis in immune cells, and its pro-apoptotic effect has been observed to counteract the anti-apoptotic effect of STAT3, establishing cross-talk between the two (236). STAT3, a member of the STAT family, is involved in the regulation of cell proliferation and survival, promoting cell proliferation and tissue repair (306). Its activation, primarily achieved through phosphorylation, enables the transmission of signals from cytokine receptors on the cell membrane to the nucleus, thereby modulating gene transcription (307). Excessive STAT3 activation often signifies increased immune activity (308). The STAT3 signaling pathway primarily contributes to immune suppression and is typically stimulated by cytokines such as interleukin-6 (IL-6), IL-10, and certain growth factors, including epidermal growth factor (EGF), transforming growth factor-β (TGF-β), and insulin-like growth factor (IGF) (309, 310). These cytokines bind to receptors on the cell membrane surface, activating JAK, which in turn promotes the phosphorylation of STAT3 and its translocation to the nucleus, forming complexes with co-activating factors, binding to target gene promoter regions and promoting transcription (311). STAT3 often modulates immune responses by inhibiting the release of pro-inflammatory factors (such as STAT3/SOCS pathway) while increasing the expression of anti-apoptotic proteins (312). In the context of interaction with CORT, the elevation of resting CORT levels due to chronic stress upregulates the expression of interleukin-10 (IL-10) and phosphorylated STAT3 (p-STAT3). Despite the significant injurious effect of CORT, apoptosis of splenic white pulp cells and increased expression of caspase-3 (composed of lymphocytes and macrophages) were observed (236). However, the use of STAT3 inhibitors exacerbated CORT-induced apoptosis of splenic immune cells, indicating a negative regulatory effect of STAT3 (236). Further investigation into the mechanism of CORT-induced apoptosis revealed that p-STAT3 regulates cell survival by inhibiting the ERS pathway rather than mitochondrial stress and death receptor activation pathways. Significant differences were observed in pro-caspase-8 and glucose-regulated protein 78 (GRP78) levels, while BCL-2, BAX, and BCL-XL levels remained unaffected. Further examination of molecules involved in the ERS pathway revealed changes in the expression of ATF6α and p-IRE1α. Although the expressions of p-JNK, pro-caspase-12 and CHOP were not upregulated, their potential involvement in regulation could not be ruled out. The lack of change in these protein levels may also result from regulation by upstream molecules, potentially influenced by differences in control variables in the study.

The elevation of CORT during chronic stress is implicated in immune cell apoptosis via ERS. This process concurrently activates anti-apoptotic pathways. Specifically, pro-inflammatory factors TNF-α and IL-1β activate the apoptotic pathway, while the anti-inflammatory factor IL-10 activates the anti-apoptotic pathway. This dual signaling in the ER triggers ERS. Increased expression of GRP78 and downstream factors, including XBP1, ATF6α and p-IRE1α leads to the UPR and protein modification errors. Elevated expression of caspase-3 and caspase-8 promotes apoptosis, while increased expression of caspase-1 and TNF-α amplifies the immune response. Additionally, activation of the JAK/STAT3) pathway leads to increased levels of phosphorylated STAT3 (p-STAT3), inhibiting the expression of pro-caspase-8, caspase-3, GRP78, ATF6α, and p-IRE1α, thus mitigating ERS and exerting anti-apoptotic effects. These findings are shown in **Figure 6**.

### 3.6 GR dysfunction

Chronic stress continuously activates the HPA axis, leading to elevated CORT levels, resulting in both the depletion of GR and a gradual loss of the HPA axis’ negative feedback capacity (313). Upon receiving this signal, cells initially undergo adaptation, prompting the overexpression of GR (65). At this juncture, both pro-inflammatory and anti-inflammatory signals are concurrently activated, highlighting the dual role of CORT, with outcomes contingent upon the gene function activated by the cell type (314). While the anti-inflammatory attributes of CORT are closely tied to normal GR function, the cytotoxic effects of CORT cannot be disregarded, as they can stimulate immune activation and the release of pro-inflammatory factors. Prolonged exposure to CORT may induce local inflammatory damage and even cell apoptosis (315–317). Persistent exposure to CORT and pro-inflammatory cytokines can diminish GR expression and prompt GR dysfunction, perpetuating inflammation (318). Consequently, aberrant GR function exacerbates the cytotoxic effects and persistent inflammation associated with CORT, concurrently diminishing cellular sensitivity to CORT, a condition known as glucocorticoid resistance (GCR) (319). Depending on tissue specificity, GCR may manifest as either sustained local inflammation or marked inhibition (314), potentially signifying prolonged exposure to CORT beyond physiological levels.

#### 3.6.1 MAPK signaling pathway

During molecular signal transmission, the strength and direction of the effect depend on both ligand concentration and receptor sensitivity. The activation of the HPA axis increases the circulating level of CORT, which extensively exerts anti-inflammatory effects by binding to the GR encoded by Nr3c1 (320). Abnormal GR function is considered an important factor in the excessive activity of inflammatory cytokines, which promotes disease development (313). Wang et al. showed that chronic immune activation during GR blockade could cause significant and persistently high levels of inflammatory cytokines (TNF-α, IL-1β, IFN-γ) in rats, alongside depressive behavior (321). Persistently high levels of TNF-α and IFN-γ overactivate the tryptophan precursor metabolizing enzyme indoleamine 2,3-dioxygenase (IDO). On the one hand, this hinders the synthesis of 5-HT (322), and on the other hand, it accelerates the decomposition of tryptophan (Try). The accumulation of its metabolites kynurenine (Kyn) and other tryptophan metabolites will trigger cellular oxidative stress damage (323, 324). ERK, p38 MAPK, and JNK are all members of the MAPK family. Upon activation through phosphorylation, they play an essential role in maintaining the fundamental signaling activities necessary for cell development (325). Numerous studies have demonstrated that the overactivation of ERK, p38 MAPK, and JNK/SAPK signals induces depressive behavior and neuroinflammation (326). This mechanism is primarily associated with decreased expression of synaptic-related genes, abnormal development of dendritic spines, increased apoptosis, and reduced expression of PSD95 (327–329). As mediators of cellular stress, the activation of the MAPK family also suppresses the expression of downstream ROS clearance genes and promotes the secretion of pro-inflammatory cytokines (330, 331). Moreover, these pathways interact with GR function. For instance, p38 regulates the phosphorylation of GR at serine sites 134 and 211 (Ser134 and Ser211), wherein activating GR phosphorylation at different sites transmits distinct signals (326). Specifically, phosphorylation of GR at Ser203 impedes nuclear translocation and reduces GR activity, whereas phosphorylation at Ser211 enhances nuclear translocation to augment GR signaling (326, 332). Zhang et al. observed that JNK activation upregulates GR phosphorylation at Ser246, consequently promoting the secretion of pro-inflammatory factors IL-1, IL-6, and TNF-α in the habenula (Hb), amygdala (Amyg), and medial prefrontal cortex (mPFC) (333). Conversely, GR activation can indirectly stimulate p38 through ROS and induce cell apoptosis via matrix metalloproteinase 13 (MMP) in certain pathways (326, 334). Thus, GR exhibits a competitive relationship with pro-inflammatory and anti-inflammatory signaling through cross-talk.

Studies have shown that chronic stress can induce GCR, resulting in inadequate control of the body’s inflammatory response to infection (245). Prolonged exposure to inflammatory cytokines such as IL-6 and TNF-α can exacerbate the expression of disease signs and symptoms, contributing to increased susceptibility to diseases. Despite the widespread expression of GRs and the myriad recognized signals, elucidating the specific mechanism of GCR remains a current challenge. Some studies propose that macrophage factor IL-1β and Th17 cytokine IL-17A may negatively impact GR function by upregulating the expression of GRβ subtypes, with GRα being the primary structure exerting effects (335, 336). This process involves the activation of the JNK and p38 MAPK signaling pathways (337, 338). In fact, a mutually inhibitory signaling pathway exists between the GR and the MAPK families. While the overexpression of GR helps in the anti-inflammatory effect of CORT, in situations where inflammation prevails, the MAPK family signals inhibit GR function (339). During the post-translational modification stage, MAPK (including JNK, P38 MAPK, and ERK) regulates GR activity by modulating the site of GR phosphorylation. Phosphorylation at sites such as Ser134, 203, and 226 inhibits GR target gene transcription (340). Additionally, GSK3β (PI3K/AKT signal) similarly impacts GR by phosphorylating Ser171 and Ser404 (341). Acetylation of GR at K494 and K495 weakens its ability to inhibit NF-κB, consequently diminishing its anti-inflammatory effect (342). These factors collectively impede the anti-inflammatory effects of CORT. Chronic stress-induced GCR can disrupt the negative feedback regulation of the HPA axis and interfere with the downstream immune system’s ability to control inflammation (343, 344). Variations in individual GR function may contribute to differences in susceptibility to cytokine-induced diseases. In terms of genetics, GR polymorphisms, such as ER22/23EK (rs6189 and rs6190), N363S (rs6195), BcII (rs41423247), and Nr3c1 gene polymorphism (Nr3c1 646 C>G), can diminish GR affinity for ligands, which then increases the susceptibility to inflammatory diseases and alters the immune milieu (340, 345, 346).

#### 3.6.2 GILZ

The signal of mutual inhibition between MAPK and GR also involves the expression of anti-inflammatory genes such as *GILZ*. The lack of *GILZ* amplifies MAPK signaling (340). *GILZ*, a gene identified in recent years, plays a crucial role in regulating the anti-inflammatory effects of GCs, with its protein expression widely contributing to anti-inflammatory effects (347). The anti-inflammatory potency of mouse GCs was observed to diminish following GILZ knockout. GILZ stands out as the earliest transcriptional target of GR (348), highlighting its significance in modulating GR activity. Current research indicates that GILZ inhibits NF-κB nuclear translocation in immune cells and interacts with activator protein-1 (AP-1) to hinder transcription (349). For instance, GILZ downregulates the expression of co-stimulatory molecules such as CD80, CD86, and MIP-1 by restraining NF-κB (113). Additionally, GILZ promotes Th2 and Treg cell phenotypes in T cells by suppressing NF-κB and activating TGF-β (350). The promotion of antigen presentation involves GILZ facilitating the process by which antigen-presenting cells display antigens to T cells, thereby initiating an immune response (351, 352). GILZ has also been found to inhibit neutrophil activation by suppressing the MAPK/ERK pathway, leading to reduced phosphorylation of ERK and p38 (353). Additionally, it controls cell proliferation and differentiation by inhibiting FOXO3 (350). In summary, given its upstream position in signaling cascades, GILZ tends to dampen immune cell activity, contributing to its anti-inflammatory properties. Changes in CORT levels during stress also influence the expression of GR. CORT in colon tissue induces an increase in both GR and GILZ expression, thereby inhibiting NF-κB activity and reducing pro-inflammatory cytokine levels such as IL-1β and TNF-α (354). Interestingly, CORT does not induce GILZ expression in brain tissue; instead, it promotes the expression of FKBP5 and SGK1 (355). Therefore, the immune-regulatory effects and expression of GILZ exhibit tissue specificity, with GILZ potentially serving as the primary mediator of anti-inflammatory effects in peripheral tissues. Given its involvement in cross-talk between signaling pathways, GILZ’s immune-regulatory mechanism may extend beyond its currently known functions, highlighting its potential as a focal point for future research.

#### 3.6.3 FKBP51

In the investigation of the CORT-GR binding structure, studies have identified an imbalance within the GR partner complex FK506 binding protein (FKBP) as a contributor to GCR (356). This imbalance is characterized by elevated levels of FKBP51 and reduced levels of FKBP52. Notably, FKBP51, encoded by the FKBP5 gene, is more susceptible to external influences and has been linked to the onset of psychiatric disorders, emerging as a key focus of current research (357). FKBP51 regulates GR activity and the function of the HPA axis by interacting with the molecular chaperone heat shock protein 90 (HSP90) (358). Despite ongoing research, the precise regulatory mechanisms governing FKBP51’s actions remain incompletely understood, generating widespread interest among researchers. Recent findings suggest that FKBP51 mediates the inhibition of AKT phosphorylation at the Ser473 site by recruiting PH domain leucine-rich repeat protein phosphatase (PHLPP), thereby leading to AKT inactivation (359). Downstream, the FKBP51/AKT signal pathway inhibits the phosphorylation activation of FOXO1 and the immunosuppressive effect of mTOR (360). Additionally, it has been observed to impede CORT-induced transcriptional regulation of GR by inhibiting GR phosphorylation (361), representing key signaling pathways for GC action. Knockout of FKBP5 leads to upregulated phosphorylation of GR at the Ser240 and Ser243 sites (362), resulting in decreased levels of pro-inflammatory cytokines such as TNF-α, IL-1β, IL-6, nerve growth factor (NGF), and brain-derived neurotrophic factor (BDNF) (363). As part of the GR complex, FKBP51 limits GR function by reducing ligand binding sensitivity. Studies have confirmed that overexpression of FKBP5 diminishes the sensitivity of GR to stress, resulting in decreased CORT secretion under stress conditions (364). Therefore, FKBP51 acts as a negative regulatory factor of GR, inhibiting GC effects, albeit lacking the ability to fully complete GR signal transduction.

In recent years, FKBP51 has emerged as having an immune-promoting effect. Studies have demonstrated that stress can modulate *FKBP5* gene expression at the epigenetic level, leading to reduced *FKBP5* methylation, particularly evident in CD4^+^ T cells and localized near chromosome 6p21.31 (365). Factors such as age, stress, and depressive phenotypes can expedite the decrease in FKBP5 methylation (358). Reduced FKBP5 methylation results in the upregulation of FKBP51 protein expression, which positively correlates with the expression of numerous pro-inflammatory genes, consequently increasing the granulocyte/lymphocyte ratio and IL-8 levels, thus driving peripheral inflammation (365). Further investigations have elucidated that *FKBP5*’s regulation of immunity hinges on NF-κB signaling. In peripheral blood mononuclear cells (PBMCs), increased FKBP51 expression promotes NF-κB signaling via the combination of NF-κB-inducing kinase (NIK) and inhibitor of kappa B kinase alpha (IKKα), thereby augmenting NF-κB signaling activity. Conversely, NF-κB signaling can induce a reduction in *FKBP5* gene methylation (resulting in increased FKBP51 expression) in immune cells. Therefore, a positive feedback loop ensues, which enhances FKBP51/NF-κB signaling and inflammation onset (365). Recent studies have shown that chronic stress-induced elevation of CORT upregulates FKBP51 expression and coincides with increased levels of pro-inflammatory factors IL-1β and TNF-α (366). While the use of FKBP51 inhibitors does not mitigate stress-induced CORT elevation, it promotes hippocampal neuronal proliferation and synaptic growth downstream, thereby mitigating social avoidance and anxiety-like behavior (367). Presently, the mechanism of FKBP51 in immune regulation remains somewhat constrained and warrants further exploration.

#### 3.6.4 CREB and FKBP51

**Figure 7:**
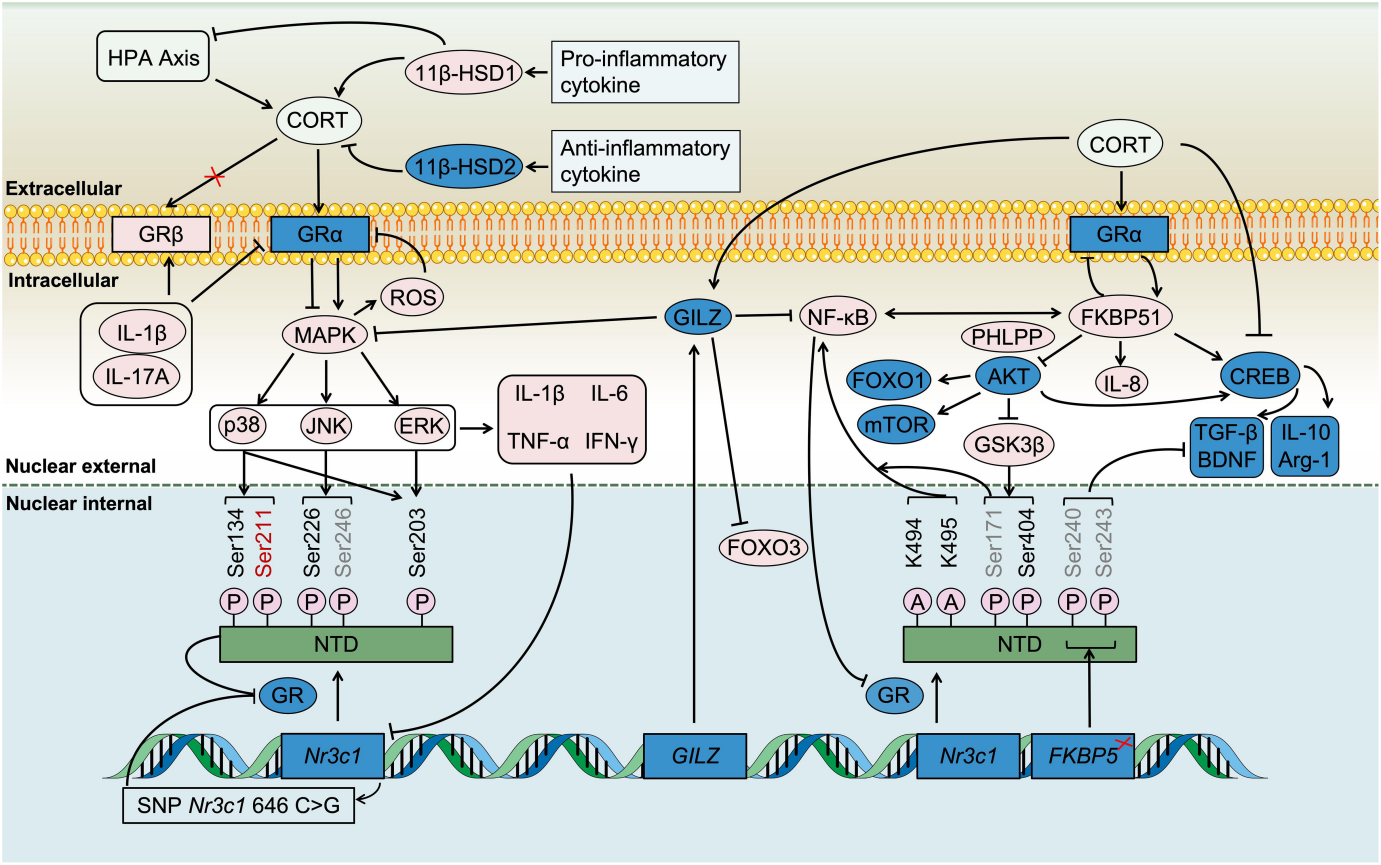
The immune regulation of CORT depends on GR signaling and 11β-HSD. CORT continuously transmits stress signals into the cell through GR, and stimulates immune cells to continuously express pro-inflammatory cytokines through the MAPK signaling pathway and FKBP51/NF-κB signaling pathway. In this scenario, on the one hand, pro-inflammatory factors change the conformation of GR (GRα to GRβ), and on the other hand, they downregulate the expression of GR. Both of these actions interfere with the normal functioning of GR and impede the transmission of anti-inflammatory signals. Due to compensatory response, the molecular partner of GR, FKBP51, is upregulated. However, elevated levels of FKBP51 increase inflammation mediated by NF-κB. In addition, it inhibits GRa. Ultimately, these effects collectively promote the progression of inflammation. Similarly, due to compensatory response, the increased expression of GILZ is to suppress the pro-inflammatory effect of CORT. However, it remains unclear whether GILZ can predominate in the complex interplay of numerous signaling pathways. In the local tissue, 11β-HSD regulates the effect concentration of CORT. 11β-HSD1 is beneficial for activating CORT and inhibiting the HPA axis, while 11β-HSD2 inactivates CORT. High expression levels of 11β-HSD1 were detected in inflammatory tissues and immune cells with a pro-inflammatory phenotype, whereas elevated levels of 11β-HSD2 were observed in immune cells with an anti-inflammatory phenotype. However, due to the persistent toxic effects of CORT, the balance is likely to shift towards the pro-inflammatory response mediated by 11β-HSD1.

In addition to ligand concentration and receptor levels, transcriptional co-regulatory proteins can also regulate CORT signaling. One such protein is CREB, a transcription factor that responds to signals from anti-inflammatory factors such as IL-4, IL-10, IL-13, TGF-β, and NGF. CREB also controls the transcriptional activation of various signaling molecules, including c-Fos, c-Jun, and BDNF, thereby facilitating neuronal cell survival, differentiation, migration, and synaptic generation (368, 369). However, CORT has been observed to inhibit CREB activation, leading to a reduction in CREB phosphorylation levels and subsequent cellular damage (215). Studies indicate that FKBP51 can regulate CREB upstream, establishing a positive feedback loop. Research conducted by Hou et al. demonstrates that CORT regulates FKBP51 and CREB in a time-dependent manner (370). Short-term treatment with CORT at concentrations of 100 nM and 1 μM promotes the formation of FKBP51/CREB protein complexes and facilitates the localization of CREB protein in the nucleus, leading to increased expression levels of both FKBP51 and CREB. However, prolonged exposure to CORT at 1 μM significantly reduces this effect. Knocking out the *FKBP5* gene directly suppresses the downstream anti-inflammatory signals of CREB in cells, resulting in decreased levels of BDNF, TGF-β, Arg-1, and IL-10. Studies have also demonstrated that activation of the CREB pathway promotes the polarization of M2 macrophages and the expression of anti-inflammatory factors, thereby inhibiting inflammatory responses (371). These findings suggest that CORT stimulation activates the FKBP51/CREB signaling pathway to adapt to stress signals. However, chronic stress can impair the cellular response mechanism. FKBP51 and CREB can directly regulate transcription by forming complexes, and there are also indirect regulatory pathways between them, such as the ERK signaling and PI3K/AKT pathway (372, 373). CREB is regulated by multiple pathways and does not act independently (215). In conclusion, both excessive and insufficient expression of the FKBP5 gene product, FKBP51, can impede the normal cellular response to CORT. The resulting effect forms a network, with the outcome dependent on the dominant signaling pathway, as illustrated in **Figure 7**.

### 3.7 11β-hydroxysteroid dehydrogenase

11β-HSD mediates the effects of CORT, which comprises type 1 and type 2 isoenzymes. 11β-HSD1 promotes GC effects by activating CORT, whereas 11β-HSD2 inactivates CORT, reducing exposure to local tissues (374). Thus, the regulatory influence of 11β-HSD on GC in various tissues has received significant attention in recent years. 11β-HSD is essential in controlling the signal transmission of CORT and GR binding in peripheral tissues. Perez et al. demonstrated that 11β-HSD1 inhibitors reduce post-stress blood CORT levels, whereas 11β-HSD2 inhibitors increase post-stress blood CORT levels. Additionally, through intraperitoneal injection and stereotactic device processing, it was found that 11β-HSD has a more pronounced regulatory effect on CORT levels in the periphery (375), underscoring the control exerted by 11β-HSD on the CORT effect. Given the intimate relationship between CORT and immunity, the regulatory role of 11β-HSD in immune inflammation has garnered attention. Sattler’s study revealed upregulated pituitary 11β-HSD1 expression in both acute and chronic arthritis mice, whereas increased hippocampal 11β-HSD1 expression was only observed in chronic inflammation, with no change in hypothalamic 11β-HSD1 expression (376), suggesting that the pituitary gland can receive feedback signals (inflammatory factors) from the periphery. Furthermore, elevated expression of 11β-HSD1 was observed in inflammatory tissues in peripheral regions (377). Increased 11β-HSD1 expression enhances the CORT effect in local tissues, highlighting the close relationship between CORT and inflammation involving 11β-HSD1 regulation. In the short term, it aids in adapting to or controlling inflammation, while in the long term, it confronts the cytotoxic-induced inflammation and pro-apoptotic effects of CORT.

Some studies have demonstrated that the use of 11β-HSD1 inhibitors can significantly mitigate the adverse metabolic pathways associated with diabetes and obesity (374). Conversely, overexpression of 11β-HSD1 in the central nervous system is more likely to dampen HPA axis activity, fostering long-term chronic inflammation and rendering the HPA axis unresponsive (376). In the periphery, 11β-HSD primarily focuses on regulating the bioavailability of CORT in various tissues (375). Similarly, Maciuszek’s study observed an increase in 11β-HSD1 expression in M1-type macrophages, while 11β-HSD2 expression was elevated in M2-type macrophages (378). This pattern may be attributed to the fact that M1 macrophages, being pro-inflammatory, require more CORT conversion to regulate inflammation by upregulating 11β-HSD1. In addition, M2 macrophages themselves secrete anti-inflammatory factors, prompting the upregulation of 11β-HSD2 to curb the excessive anti-inflammatory effect of CORT. Young’s cell experiments indicated that 11β-HSD1 downregulated the secretion of IL-1β and IL-6 by catalyzing the generation of CORT, thereby inhibiting the pro-inflammatory response mediated by NF-κB activation (379). This suggests that, apart from HPA axis activation to produce CORT, 11β-HSD1, as an indirect regulatory pathway, promotes the local production of CORT to adapt to the local environment. Additionally, Du’s research proposes that exercise training boosts the expression of 11β-HSD1 in the lungs of obese mice, aiding in the activation of local CORT and inhibition of pneumonia (380). Therefore, 11β-HSD also plays a crucial role in mediating the immune regulation of CORT under stress and constitutes an integral component of its immune regulatory mechanism, as depicted in **Figure 7**, warranting further exploration.

## 4 Conclusion

CORT is closely related to immunity and is influenced by multiple signals. During acute stress, it surges rapidly, aiding in rapid stress responses and inducing immunosuppression through its potent anti-inflammatory properties, which are essential for maintaining internal homeostasis. Subsequent negative feedback from the HPA axis reduces CORT levels. However, chronic stress results in a gradual increase in CORT, continuously activating the immune system. Prolonged stress leads to elevated CORT levels, causing abnormal expression of GR and 11β-HSD in various circulating tissues, disrupting CORT’s anti-inflammatory effects and impeding HPA axis negative feedback, perpetuating immune system activation and fostering chronic systemic inflammation. As circulating CORT levels rise, its cytotoxic effects intensify, exacerbating internal inflammation and triggering cellular autonomous death processes, impairing tissue function. Thus, immune suppression arises from excessive immune system activation and consumption, highlighting the complex relationship between CORT, immune function, and stress duration, necessitating further investigation into its mechanisms.

**Figure 8:**
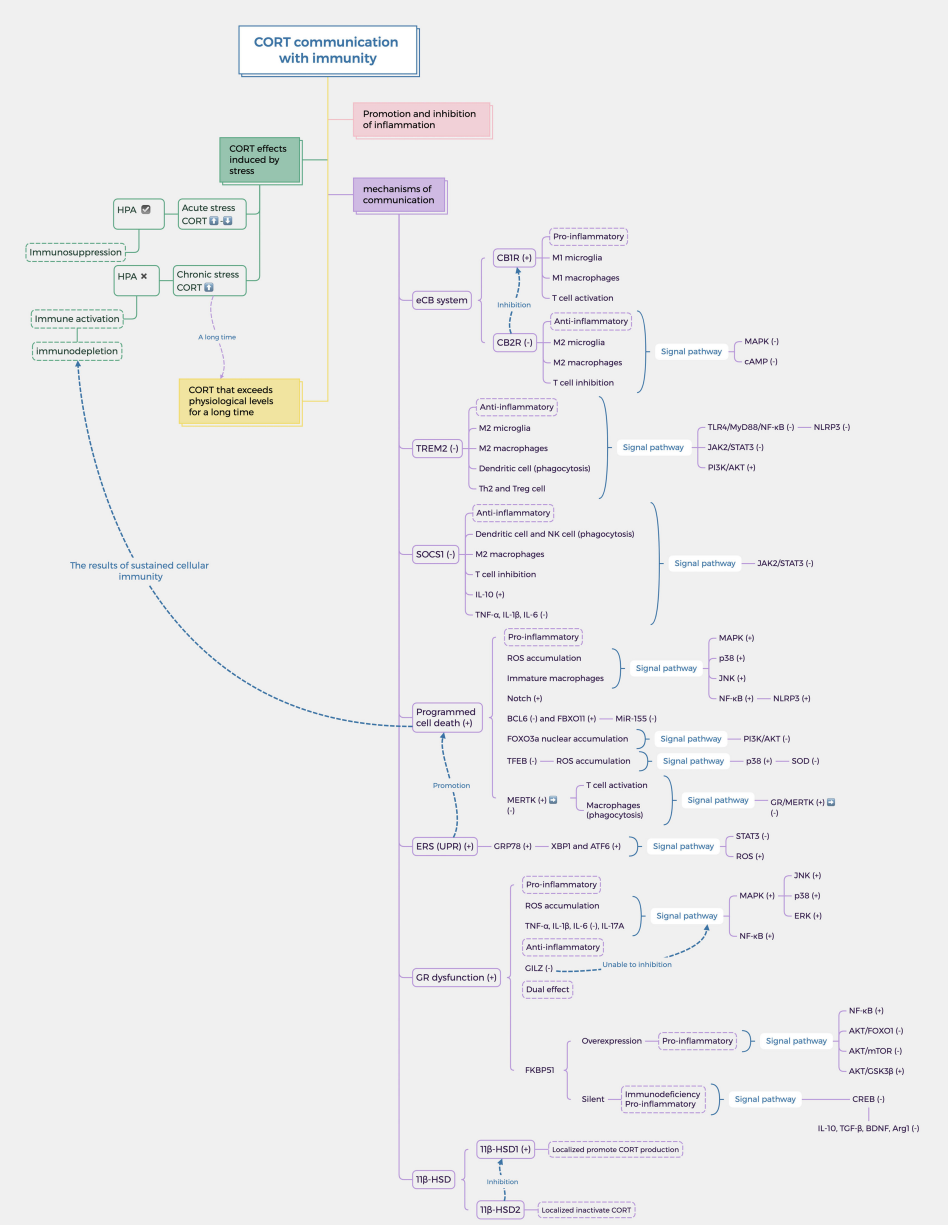
Summary of interaction mechanism between CORT effect and immunity.

CORT functions as a GC molecule in the bloodstream and it can affect various tissues across the body. Its interaction with the immune system primarily involves the exchange of inflammatory cytokines and the signal transduction of cellular function within immune cells. During chronic stress, elevated CORT poses a challenge to immune cells. Initially, resting immune cells tend to polarize towards pro-inflammatory phenotypes, releasing pro-inflammatory and chemotactic factors to recruit assistance, often through MAPK, NF-κB, and other signaling pathways. This immune activation unavoidably consumes energy and metabolites. To prevent immune failure and cell death, signals that maintain homeostasis and promote cell survival, such as proliferation, differentiation, and maturity, are simultaneously activated. These signals operate through pathways like PI3K/AKT, cAMP/CREB, STAT3, Nrf2, and others. In the cytoplasm, signal transduction affects gene expression, transcriptional strength, and protein translation and modification, such as the expression of genes like GILZ and SOCS, the transcription of MiR-155 and TFEB, the expression of TREM2 and TAM, and the synthesis of eCB. In the communication between CORT and immune cells, these signals promote external anti-inflammatory responses and internal inhibition of intracellular stress signals. However, sustained high levels of CORT override these protective responses one by one, favoring pathways leading to injury, such as apoptosis signaling triggered by NRPL3, inadequate synthesis of ROS decomposition, nuclear accumulation of FOXO3a, and sustained activation of the Notch pathway and ERS-IRE1/XBP1 signaling pathway, all contributing to cell death. Therefore, immune balance is disrupted, leading to the progression of inflammation into disease. Pro-inflammatory symptoms signify continuous immune function until immune depletion occurs. Additionally, during the CORT process, the number and structural abnormalities of GR (excessive beta structure and insufficient alpha structure) and imbalanced expression of 11β-HSD (excessive 11β-HSD1 and insufficient 11β-HSD2) prevent the anti-inflammatory effects of CORT, contributing to GC resistance in immunotherapy. As shown in **Figure 8**.

It is essential to acknowledge that epigenetic changes and genetic polymorphisms influenced by environmental factors and lifestyle habits are potential contributors to the effects of CORT on immune regulation. Moreover, it is evident that immune regulation balance is ubiquitous, reflecting the intricate interplay between various factors and pathways. Recent hot research topics include macrophage and microglia polarization into M1/M2 phenotypes, T cell expression balance (Th1/2 and Th17/Treg), cannabinoid receptors CB1R and CB2R, immune cell membrane receptors TREM1 and TREM2, chaperone proteins FKBP51 and FKBP52, and enzymes 11β-HSD1 and 11β-HSD2. The equilibrium of these immune substances is critical for maintaining normal physiological functions. Prolonged exposure to external stressors, such as chronic family and social stress, fundamentally disrupts the immune balance mediated by CORT.

**Figure 9:**
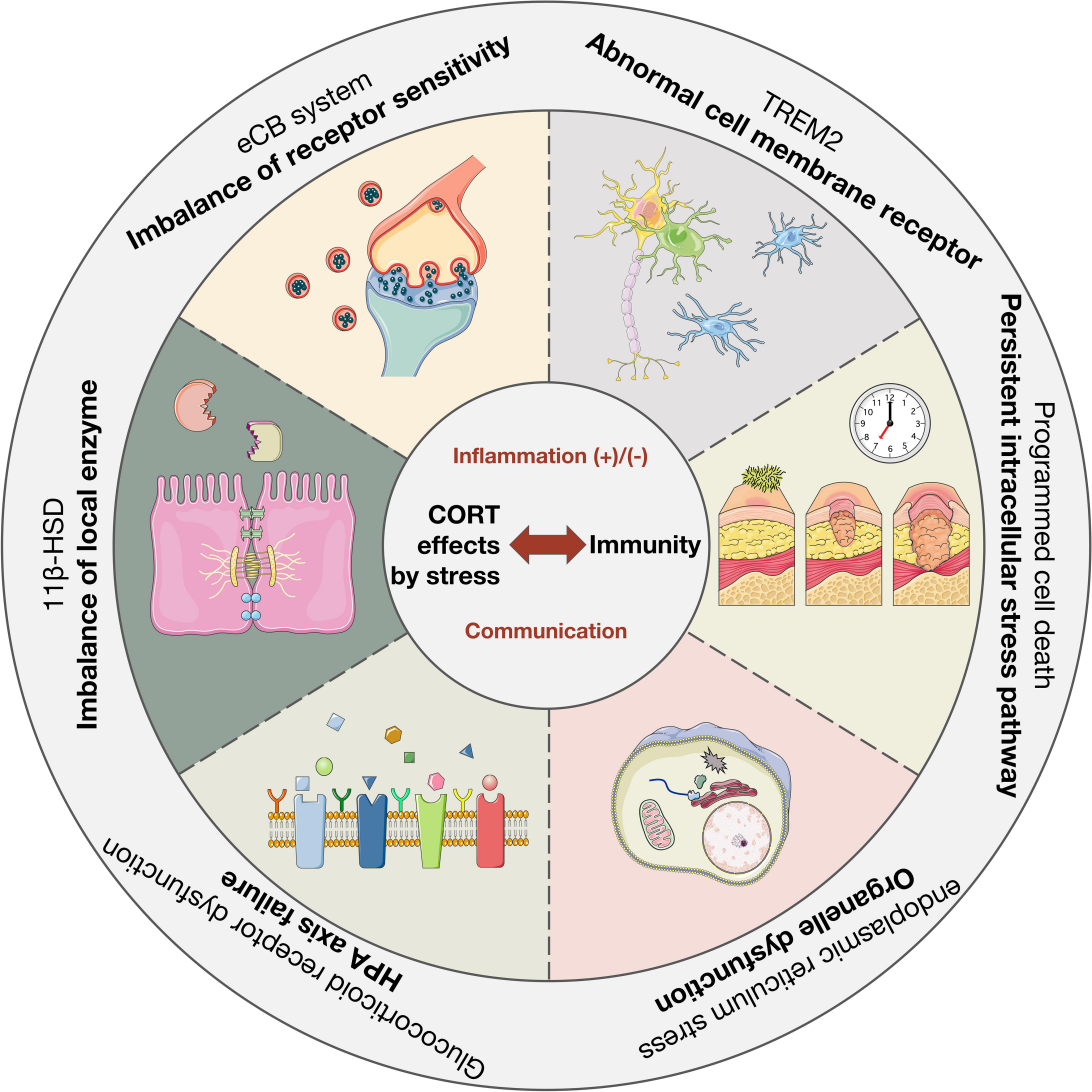
The way between CORT effect and immunity regarding inflammation.

In conclusion, this review discusses the diverse and interconnected pathways between CORT and immune regulation. As shown in **Figure 9**. A comprehensive understanding of these regulatory mechanisms is vital for recognizing the close relationship between stress, emotional disorders, immunity, and inflammation, providing new avenues for treatment. Several key targets and immune regulatory proteins that are closely associated with CORT may serve as potential clinical biomarkers for the early screening of diseases. The identification and utilization of these biomarkers could benefit the health management of stress-related diseases, enabling more timely and effective interventions. Meanwhile, elucidating the underlying mechanisms and identifying key targets is highly advantageous for the development of novel therapeutic strategies. For instance, the discovery of small molecule drugs targeting specific pathways, the development of immune modulators, and the application of gene therapies all hold great promise. These advancements may pave the way for innovative treatment approaches for stress-related diseases, ultimately enabling more precise and efficient therapeutic interventions. Above all things, it is imperative to prioritize addressing stressors to prevent sustained elevation of CORT, thereby safeguarding immunity.

## 5 Limitations

While the role of CORT has been extensively studied, its intricate connection with the immune system is also gaining increasing attention. Nevertheless, there are still several questions that remain unclear at present. Specifically, (1) it is widely accepted that CORT levels serve as a biomarker reflecting stress conditions. Current research is generally categorized into two types: acute stress and chronic stress, both of which are used to observe the relationship between CORT and immune phenotypes. However, during the stress process, organisms exhibit physiological responses of adaptation and compensation. Whether immune activation during this period is beneficial for functional enhancement of tissues or immediately causes inflammatory damage remains unclear and requires more rigorous phenotypic evidence. (2) In current research, there are various methods for simulating stress. Although CORT levels typically increase in response to stress, different stress paradigms may lead to divergent outcomes in terms of CORT effects. This is also one of the reasons why some studies report opposing results. A more detailed comparative study could be conducted to elucidate these differences. (3) With the cessation of stress, there is potential for the repair of immune activation and chronic inflammation. And persistent elevation of CORT levels remains a primary cause of irreversible inflammatory damage. However, the exact duration of stress required to trigger such immune damage is currently unclear. (4) CORT levels within the physiological range is intricately linked to innate immune function. It is evident that there are inherent variations in CORT levels among individuals. These differences may influence how individuals adapt to and respond to stress, leading to distinct outcomes. Among them, CORT may exhibit diverse patterns of effect. This variability is likely one of the reasons why different individuals exhibit diverse pathological characteristics when exposed to stress. Further clinical research, combined with in-depth basic research, is essential to explore and elucidate these differences. (5) Immune cells are ubiquitous, and the majority of immune cells and tissue cells express GR. Given that CORT is a glucocorticoid, the immune damage caused by stress is systemic in nature. It is not confined to the central nervous system or related to mental illness alone. Therefore, CORT may exert distinct immune effects in different types of tissues (sites). For instance, 11β-HSD exhibits varying expression patterns across different tissues, thereby mediating divergent immune responses. This area certainly warrants more in-depth exploration. (6) The interaction mechanisms between recently discovered key immune regulatory proteins and stress-related CORT remain to be elucidated. Clarifying these mechanisms is also one of the promising avenues for exploring new therapeutic targets. (7) Based on the current understanding of CORT’s role, its significance extends to certain unique environments. For instance, in the mechanisms underlying intercellular competition, there is a potential for CORT to be involved. As an example, atypical immune cells such as iT cells may have their differentiation or immune regulatory direction influenced by CORT. Further research is needed to elucidate these mechanisms.

Currently, there is a relatively comprehensive understanding of the pathways through which CORT interacts with the immune system. Based on current research trends and hot topics, it is anticipated that more mechanisms of CORT will be uncovered in the future. As a key marker of stress, CORT holds significant research value across multiple system diseases. Moreover, identifying additional targets of CORT would be highly beneficial for the development of new small-molecule drugs.

## Glossary

AA: Arachidonic acid
AC: Adenylate cyclase
ACS: Apoptotic speck-like protein containing a caspase recruitment domain
ADAR1: Double-stranded RNA adenylate deaminase antibody 1
AEA: Anandamide
AgRP: Agouti-related protein
AKT: Protein kinase B
AP-1: Activator protein-1
Arg-1: Arginase-1
ATF6: Activating transcription factor-6
ATM: Ataxia-telangiectasia mutated proteins
BAX: BCL2-Associated X
BCL-2: B-cell lymphoma-2
BDNF: Brain-derived neurotrophic factor
cAMP: cyclic adenosine monophosphate
CBR: Cannabinoid receptor
CCR: C chemokine receptor
CHOP: C/Ebp-Homologous Protein
CIC: Circulating immune complex
CREB: cAMP response-element protein
CORT: Corticosterone
COX: Cyclooxygenase
CRH: Corticotropin releasing hormone
CTL: Cytotoxic lymphocyte
CUMS: Chronic unpredictable mild stimulation
CYP450: Cytochrome P450
DAGL: Diacylglycerol lipase
DAP12: DNAX-activating protein of 12 kDa
DC: Dendritic cell
DDR: DNA damage repair
DNA: Deoxyribonucleic acid
EGF: Epidermal growth factor
EREG: Epidermal regulatory factor
ERK: Extracellular regulated protein kinases
ERS: Endoplasmic reticulum stress
eCB: Endocannabinoids
FAAH: Fatty acid amide hydrolase
Fas: TNF receptor superfamily, member 6
Fasl: Fas ligand
FGF: Fibroblast growth factor
FKBP: FK506 binding protein
FOXO: Forkhead box O
GABA: γ- aminobutyric acid
GC: Glucocorticoid
GCR: Glucocorticoid resistant
G-CSF: Granulocyte-colony stimulating factor
GILZ: Glucocorticoid induced leucine zipper
GM-CSF: Granulocyte macrophage-colony stimulating factor
GR: Glucocorticoid receptor
GRP78: Glucose regulatory protein 78
GSDMD: Gasdermin D
GSH: Glutathione
GSK: Glycogen synthetase kinase
GST: Glutathione transferase
HIP: Hippocampus
HPA: Hypothalamic-pituitary-adrenal
HSP: Heat shock protein
ICOS: Inducible T cell costimulator
IDO: Indoleamine 2,3-dioxygenase
IFI-16: Interferon induced nuclear protein-16
IFN: Interferon
IFNGR2: Interferon Gamma Receptor 2
IGF: Insulin-like growth factor
IKK: Inhibitor of kappa B kinase
IL: Interleukin
iNOS: Inducible nitric oxide synthase
IP-10: Inducible protein-10
IRBP: Interphotoreceptor retinal binding protein
IRE1: Inositol requires enzyme 1
JAK: Janus kinase
JNK: c-Jun N-terminal kinase
LC: Locus coeruleus
LDHA: Lactate dehydrogenase-A
LIF: Leukemia inhibitory factor
LIX: Lipopolysaccharide-inducible CXC chemokine
LOX: Lipoxygenase
LPS: Lipopolysaccharide
LXA4: Lipoxygen-A4
MAGL: Monoacylglycerol lipase
MAIT: Mucosa-associated invariant T
MCP-1: Monocyte chemoattractant protein-1
M-CSF: Macrophage-colony stimulating factor
MDA-5: Melanoma differentiation associated gene-5
MDD: Major depressive disorder
MIP-1: Macrophage inflammatory protein-1
MHC: Major histocompatibility complex
MLKL: Mixed lineage kinase domain-like
mTOR: mammalian target of rapamycin
MyD88: Myeloiddifferentiationfactor 88
MMP: Matrix metalloproteinase
NAPE-PLD: N-acylphosphatidylethanolamine-hydolyzing phospholipase D
NE: Norepinephrine
NF-κB: Nuclear factor-κB
NGF: Nerve growth factor
NICD1: Notch1 intracellular domain
NIK: NF- κB-induced kinase
NK: Natural killer
NKT: Natural killer T
NLRP3: NOD-like receptor thermal protein domain associated protein-3
NO: Nitric oxide
NPY: Neuropeptide Y
NPY1R: Neuropeptide Y receptor-1
PAF: Platelet activating factor
PBMC: Peripheral blood mononuclear cell
PD: Programmeddeath
PG: Prostaglandin
PHLPP: PH Domain Leucine-rich Repeat Protein Phosphatase
PI3K: Phosphatidylinositol 3-kinase
PKR: Double-stranded RNA-dependent protein kinase
PLCγ2: Phospholipase Cγ2
PRR: Pattern recognition receptor
PVN: Paraventricular hypothalamic nucleus
P2X7: Purinergic 2X7
RA: Rheumatoid arthritis
RIPK3: Receptor interaction serine threonine protein kinase 3
ROS: Reactive oxygen species
RTK: Receptor tyrosine kinase
SAPK: Stress-activated protein kinase
SNS: Sympathetic nervous system
SOCS: Suppressor of cytokine signaling
SOD: Superoxide dismutase
STAT: Signal transducers and activators of transcription
TCR: T cell receptor
TFEB: Transcription factor EB
TGF-β: Transforming growth factor-β
TLR: Toll-like receptor
TNF: Tumor necrosis factor
TREM2: Triggering receptor expressed on myeloid cells-2
TREX1: three prime repair exonuclease 1
UPR: Unfolded protein response
VEGF: Vascular endothelial growth factor
XBP1: X-box binding protein 1
XO: Xanthine oxidase
ZBP1: Z-DNA Binding Protein 1
2-AG: 2-arachidonoylglycerol
3β-HSD: 3β-hydroxysteroid dehydrogenase
5-HT: 5-hydroxytryptamine
11β-HSD: 11β-hydroxysteroid dehydrogenase.
